# Hydrolysis and depletion of phosphatidylglycerol at peak murine acute lung injury

**DOI:** 10.1016/j.jlr.2026.101068

**Published:** 2026-06-27

**Authors:** Brandon Baer, Michael C. Seeds, Michael V. Novotny, Matthew J. Satusky, Christopher M. Lloyd, Barbara Olmeda, Jesus Perez-Gil, R. Duncan Hite

**Affiliations:** 1 Division of Pulmonary, Critical Care and Sleep Medicine, University of Cincinnati College of Medicine, Cincinnati, OH, USA; 2 Wake Forest Institute for Regenerative Medicine, Wake Forest School of Medicine, Medical Center Blvd, Winston-Salem, NC, USA; 3 Department of Inflammation and Immunity, Lerner Research Institute, Cleveland Clinic, Cleveland, OH, USA; 4 Renaissance Computing Institute, University of North Carolina at Chapel, Chapel Hill, NC, USA; 5 Departamento de Bioquimica y Biologia Molecular, Facultad de Biologia, Universidad Complutense, Madrid, Spain; 6 Instituto de Investigación Hospital 12 de Octubre (imas12), Madrid, Spain

**Keywords:** acute respiratory distress syndrome, acute lung injury, pulmonary surfactant, phosphatidylglycerol, secretory phospholipase A_2_, surface tension

## Abstract

Anionic phospholipids are essential for surfactant function and phosphatidylglycerol (PG) is the most abundant. Increased secretory phospholipase A2 (sPLA_2_) and disproportionate depletion of PG is consistently observed in patients with acute respiratory distress syndrome (ARDS), leading to speculation on their potential relationship. We hypothesized that a non-lethal murine model of acute lung injury (ALI) would demonstrate a temporal association between lung inflammation, surfactant dysfunction, PG depletion, and sPLA_2_ activity at peak ALI. ALI was induced in BALB/c mice by intratracheal lipopolysaccharide (IT-LPS). Markers of ALI, surfactant (function, recovery, and composition), and sPLA_2_ activity were assessed over a 240-h period encompassing peak ALI and resolution. During peak ALI, a separate cohort of IT-LPS mice received IT-varespladib, an sPLA_2_ inhibitor, with surfactant outcomes analyzed 4 h later. IT-LPS induced features of ALI, including immune cell influx, protein leak, histological injury, and respiratory dysfunction, which peaked at 48–96 h and resolved by 240 h. Impaired surfactant function at peak ALI was associated with increased sPLA_2_-mediated PG hydrolysis and reduced PG content. All changes were absent post-ALI resolution. Varespladib reduced alveolar sPLA_2_ activity, increased surfactant PG, and improved surfactant function compared to untreated LPS controls. At peak ALI after IT-LPS, depletion of PG is synchronously related to surfactant dysfunction and increased sPLA_2_ hydrolysis. Pharmacologic inhibition of sPLA_2_ at peak ALI improves PG content and surfactant function. Additional substudies to fully examine their relationships and mechanisms are warranted.

Acute respiratory distress syndrome (ARDS) is a life-threatening condition associated with high mortality and substantial morbidities, including prolonged mechanical ventilation, slow recovery of respiratory function, and significant healthcare costs (1, 2, 3). ARDS can arise from a range of direct or indirect pulmonary insults, such as sepsis, pneumonia, trauma, and pancreatitis. As a part of the host response to these initiating events, multiple inflammatory pathways become activated, with mediators originating from both systemic and pulmonary cellular sources. This inflammatory cascade contributes to injury of the pulmonary vasculature and alveolar epithelium, as well as degradation and functional impairment of pulmonary surfactant (1, 2, 4, 5, 6).

Pulmonary surfactant is a complex mixture of phospholipids (80%–90%), neutral lipids (5%–10%), and specialized proteins (5%–10%) that principally serve to lower surface tension and maintain patency of alveoli and small airways (7, 8, 9). Among the four surfactant proteins, the two hydrophobic cationic surfactant proteins, surfactant protein B (SP-B) and surfactant protein C (SP-C), provide the principal interactions with the surfactant phospholipids that account for the surface tension-lowering activity of pulmonary surfactant (10, 11, 12). The most abundant surfactant phospholipid is phosphatidylcholine (PC; 80%), and dipalmitoyl-PC provides key contributions through stabilization of lipid film during maximum compression (exhalation). The second most abundant is phosphatidylglycerol (PG; 10%–12%), an anionic phospholipid that plays an equally essential role in the surface tension-lowering activity through its interactions with SP-B (13, 14, 15, 16, 17). Enzymatic degradation of surfactant phospholipids is a clinically relevant inflammatory mechanism contributing to surfactant dysfunction in ARDS (6, 18, 19, 20, 21). Many changes have been observed in the surfactant composition of bronchoalveolar lavage (BAL) fluid from patients with ARDS, including a consistently disproportionate depletion of PG (21, 22, 23, 24, 25, 26). The functional significance of this isolated change is unknown and not fully explored.

In addition to surfactant changes, BAL analysis in ARDS demonstrates increased activity of secretory phospholipase A2 (sPLA_2_) (20, 21, 27, 28, 29, 30, 31). The family of sPLA_2_ enzymes includes several isoforms in mammals and is characterised by calcium dependence, extracellular phospholipid hydrolysis at the *sn*-2 position (32), and upregulation as part of the innate immune response against microbial pathogens (33, 34). Multiple isoforms have also been localized to the lungs in humans (27, 35, 36, 37, 38, 39, 40). As a result, surfactant phospholipid films at the air-liquid interface and phospholipid aggregates within the alveolar space likely serve as “innocent bystander” substrates for sPLA_2_ released into the alveolus as part of the immune response (21, 27, 30, 32, 41).

In our previous in vitro studies focused on sPLA_2_-mediated surfactant dysfunction, depletion of PG was demonstrated to be a more sensitive and functionally significant mechanism of surfactant dysfunction than the generation of enzymatic byproducts, lysophospholipids, and free fatty acids in ARDS (41, 42). Multiple pre-clinical animal models of acute lung injury (ALI) have demonstrated increased sPLA_2_ activity and changes in surfactant, but did not include synchronous analyses of PG content, PG hydrolysis activity, and surfactant dysfunction. As such, we hypothesized that a transient, non-lethal model of ALI could affirm an association between localized lung inflammation, impaired surfactant dysfunction, increased sPLA_2_ activity, and PG depletion. Once affirmed, the model might also subsequently serve as the basis on which multiple sub-studies could be conducted to directly examine and define the mechanistic relationships between PG hydrolysis, PG depletion, impaired surfactant function, and their potential for therapeutic reversibility.

## Materials and Methods

### Animals

All procedures were reviewed and approved by the Wake Forest School of Medicine, the Cleveland Clinic Lerner Research Institute, and University of Cincinnati College of Medicine Institutional Animal Care and Use Committees. All experiments were performed using wild-type, male, BALB/c mice (8–10 weeks old) purchased from Jacksons Laboratory. Animals were housed in the University’s animal facilities with ad libitum access to food and water.

### Lipopolysaccharide-induced ALI

To induce ALI, mice were first anesthetized using intraperitoneal ketamine:xylazine:acetylpromazine (110, 5, and 1.7 mg/ml, respectively) at a dose of 2.8 μg/g of mouse body weight. Post-anesthesia, mice were intubated with a 20-gauge flexible IV catheter and intratracheally instilled with 30–40 μl of lipopolysaccharide (LPS, *Escherichia coli*; 0111:B4; Sigma-Aldrich, Burlington, MA; 9 mg/kg) in sterile saline. Sham mice underwent the same procedure with the instillation of an equivalent volume of sterile saline. Once recovered, mice were returned to the animal facility until their respective endpoints over a 240-h time course.

### Harvest time points

Mice were divided into separate groups based on harvest time points and to fulfill all clinically relevant measurements of an ALI model as recommended by the 2022 American Thoracic Society workshop report (43). The first group of mice was utilized to determine the initiation, peak, and recovery time course of LPS-induced inflammation and edema, with BAL fluid being collected at 0, 4, 24, 48, 96, 168, and 240 h post intratracheal instillation. Time course of illness severity was also determined through changes in body weight, with weights being recorded at baseline (0 h) and across all harvest time points. After completion of these initial studies, all subsequent cohorts were harvested at fewer time points, with focus on time points corresponding to peak injury (48 or 96 h) and resolution (240 h). The cohorts used to evaluate surfactant function, surfactant phospholipid content, sPLA_2_ activity, and lung mechanics were measured at 0, 48, 96, and 240 h. The cohort used to evaluate histological lung injury was measured at 0, 48, and 240 h.

### Bronchoalveolar lavage

Whole lung lavage was performed after euthanasia through a secured endotracheal tube using three 1 ml aliquots of saline that were pooled on ice for each mouse. The collected fluid was labelled as bronchoalveolar lavage (BAL) and the cellular fraction of the BAL was isolated into a pellet using centrifugation (700*g* for 10 min). Cell pellets were resuspended in 1 ml of phosphate-buffered saline, with counts performed on a hemocytometer for total inflammatory cells or a Cytospin slide for cell differential counts using Diff-Quick (Thermo-Fisher) staining. The resulting cell-free BAL was used to measure surfactant endpoints as described below.

### Surfactant fractionation

Cell-free BAL supernatant was further centrifuged at 40,000*g* for 20 min to obtain a large aggregate (LA; active surfactant) pellet, which was washed twice with saline by ultracentrifugation (40,000*g*), before being resuspended in 100 μl of saline. The resulting supernatant was labelled as BAL supernatant and used to evaluate BAL protein and phospholipase activity. In both the cell-free BAL (total) and LA subfraction, phospholipid content was measured via lipid phosphorous as described previously (44). Protein content was measured using a BCA assay (ThermoShandon-Pierce).

### Surfactant function

Biophysical surfactant function was quantified using ex vivo surface tension measurements on a pulsating bubble surfactometer (PBS; General Transco Seminole) (21, 45). An aliquot of isolated LA was resuspended to 1 mg/ml phospholipids in 150 mM NaCl, 5 mM CaCl_2_ 5 mM Tris solution. To achieve triplicate runs on the PBS (40 μl volume per run) per sample at this concentration, the LA from multiple mice (N = 3) in each treatment group were pooled for each data point. Briefly, samples were preheated to 37°C for at least 5 min before being pulsated at 20 pulses/min for 20 min. Surfactant function data were presented as the minimum surface tension reached during the 20-min measurement period.

### Surfactant phospholipid composition

To evaluate the phospholipid composition, an aliquot (30 nmol of phospholipid) from the pooled LA samples was organically extracted (46), dried under nitrogen gas, and re-suspended in chloroform. The major individual phospholipid groups that comprise pulmonary surfactant were identified using previously published methods (47). Briefly, the extracted samples were separated using high-performance liquid chromatography (HPLC) over Kromasil Silica (5 μm, 100A, 4.6 mm × 150 mm) on a Waters HPLC system. Peaks of each lipid were detected with a Sedere Sedex55 ELSD, integrated, and the areas under the curve were used to determine lipid amounts compared to known standards of each phospholipid purchased from Avanti Polar Lipids. Specifically, concentrations curves of 0.25–4 nmol were generated using dipalmitoylphosphatidylglycerol (DPPG; SKU 840455P), dipalmitoylphosphatidylcholine (DPPC; SKU 850355C), lysophosphatidylcholine (LPC; SKU 855675C), phosphatidylethanolamine (PE; SKU: 850705P), phosphatidylinositol (PI; SKU: 840042C; Liver, Bovine), sphingomyelin (SPH; SKU: 860061C; Egg, Chicken), and lysophosphatidylglycerol (LPG; SKU 858125C). Before analysis, all unknown samples were supplemented with a fixed amount of phosphatidylbutanol (SKU: 860202C), which served as an internal dilution standard to facilitate the quantification of PC. Data are presented as both the percentage of total phospholipids in the LA and the absolute amount of each phospholipid recovered.

### Secretory phospholipase activity

To measure sPLA_2_ activity in BAL (supernatant post separation of LA), hydrolysis of Survanta (Abbvie Inc.) was assessed at a concentration of 2.0 mg/ml. Briefly, Survanta was labeled with known amounts of [3H]-dipalmitoyl-PG (10 μCi/μmol, ∼30,000 dpm/sample) or [3H]-dipalmitoyl-PC (100 μCi/μmol, ∼100,000 dpm/sample; American Radiolabeling Company, St. Louis, MO), using repetitive vortexing and confirmation with sucrose gradients, as previously described (41, 48), before being incubated for 2 h with BAL supernatant. Radiolabeled 1-[3H]-glycerol-PG was obtained from [3H]-glycerol-labeled *Escherichia coli* following previously described methods (49). The labeled PG was purified through lipid extraction and subsequent separation by thin-layer chromatography. After assessing radioactivity and phosphorus content, samples were stored in chloroform solution. Hydrolysis was determined by separation of intact labeled phospholipids from free fatty acid ([3H]-PC) or free lyso-[3H]-PG fractions using thin layer chromatography for separation and a scintillation counter (48, 50). Results were reported as the percentage of the total label found in the free hydrolyzed form (PG or PC).

To determine whether phospholipid hydrolysis in the BAL supernatant was attributable, at least in part, to altered sPLA_2_ activity rather than other PLA_2_ subtypes (e.g., cytosolic or lysosomal), the sPLA_2_ PG hydrolysis assay described above, was repeated under modified conditions. Specifically, PG hydrolysis was measured following ex vivo treatment of BAL supernatant with either a calcium-chelating agent (EGTA; 5 mM), sPLA_2_ inhibitor (varespladib; 50 μm), or an equivalent volume of saline. These experiments were performed using BAL supernatant isolated from LPS-instilled mice, harvested at the 48-h time point.

### Lung mechanics

Mice were sequentially anesthetized, endotracheally cannulated, and paralyzed (Pancuronium Bromide; 0.8 mg/kg) prior to lung function measurements. Briefly, lung mechanics were assessed using a flexiVent system (SCIREQ, Montreal, Canada), measuring parameters including compliance, resistance, elastance, and hysteresis (51, 52). Mechanical ventilation was delivered with a tidal volume of 10 μl/g starting body weight, a respiratory rate of 150 breaths/min, and a positive end-expiratory pressure of 3 cm H_2_O.

### Lung histology

Mice were euthanized prior to the insertion of an endotracheal tube into their trachea. Animals were perfused with phosphate buffered saline via the pulmonary artery, while their lungs were simultaneously inflated and deflated using a respiratory bulb. Next, the lungs were inflated with Tissue-Tek® optimal cutting temperature compound (OCT; Sakura Finetek) through the trachea (53). Lungs were then excised, snap frozen in OCT filled cassettes using liquid nitrogen, embedded in paraffin, and sectioned. For histological assessment of injury scores, six non-overlapping fields were imaged across both lungs at 20x magnification. Scoring was performed in a blinded fashion using a five-point scale across four parameters: (*1*) inflammation, (*2*) septal thickening, (*3*) edema, and (*4*) red blood cells (RBC) in the alveolar space (54). Scores for each parameter were averaged across the six images to generate a total lung injury score. Across all cohorts and time points, mice were euthanized using anesthetic overdose and cervical dislocation.

### Lung SP-B and SP-C

Protein levels of SP-B and SP-C in BAL were quantified through Western blot as described previously (55). Briefly, BAL samples were lyophilized and rehydrated in saline buffer, and phospholipid content was quantified as described previously (56). For SP-B, samples were combined with loading buffer under non-reducing conditions, while for SP-C, the loading buffer was supplemented with 4% (w/v) β-mercaptoethanol. Samples were heated for 10 min at 95°C, before loading 10 μg of phospholipid in 16% acrylamide gels and performing SDS-PAGE. Proteins were transferred to a PVDF membrane (Bio-Rad Laboratories, Inc.) by wet electroblotting at 300 mA for 60 min. Membranes were then blocked with PBS-Tween containing 5% (w/v) skimmed milk before being incubated overnight at 4°C with 1:5000 anti-human SP-B antibody (Rabbit; WRAB-48604; Seven Hills Bioreagents) or 1:5000 anti-human SP-C antibody (Rabbit; WRAB-76694; Seven Hills Bioreagents). The secondary HRP linked anti-IgG antibody (Swine; P021702-2; Agilent Technologies) was used at 1:5000, and bands were visualized after incubation in ECL solution (Merck Millipore). Densitometric analysis was performed with Quantity One software (Bio-Rad Laboratories). Protein expression was presented as percent of baseline, being compared to untreated mice (0 h).

### In vivo varespladib treatment

To more specifically characterize the relationship between sPLA_2_-mediated hydrolysis of surfactant lipids, surfactant dysfunction, and PG depletion, an additional cohort of LPS instilled mice were utilized. At 48 h post LPS, mice were administered intratracheal Varespladib (5.7 μg/mouse; Sigma Aldrich) and compared to untreated LPS controls. Mice were then harvested 4 h later, and all surfactant endpoints were performed using the methods described above.

### Statistical analysis

All mice utilized for this study were included resulting in a total of 323 mice being utilized: 44 untreated or time 0 mice [33 mice for BAL/pooled for surfactant analysis, 3 mice for histology, and 8 mice for lung mechanics], 123 sham mice [103 mice for BAL/pooled for surfactant analysis, 3 mice for histology, and 17 mice for lung mechanics], and 156 LPS mice [96 mice for BAL/pooled for surfactant analysis, 6 mice for histology, 30 mice for lung mechanics, and 24 mice for BAL/pooled for surfactant analysis in the in vivo Varespladib (15 untreated LPS mice and 9 Varespladib treated LPS mice)]. Data analysis was performed using GraphPad Prism (version 10.6.1) and are presented as mean ± standard error of the mean in figures and mean ± standard deviation in the tables. Except for minimum surface tension measurements and HPLC, where each data point represents a pooled collection of LA samples from multiple mice in the same treatment group, each data point corresponds to an individual mouse. Statistical differences between two groups were assessed in figures using a Mann-Whitney test and unpaired *t* test in tables. Statistical differences between three or more groups were evaluated using either one-way ANOVA followed by Dunnett's multiple comparisons test or two-way ANOVA followed by Sidak’s multiple comparisons test. Statistical significance was defined as a *P*-value of less than 0.05.

## Results

### Time course of acute lung injury

**Fig. 1:**
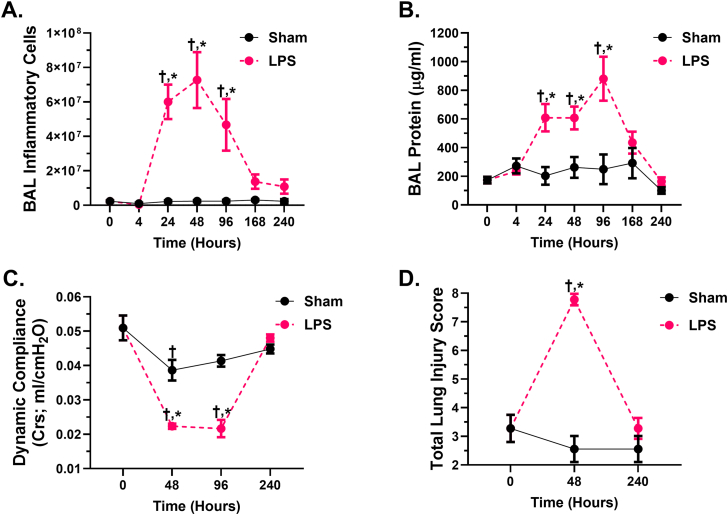
Parameters of acute lung injury induced by IT LPS (9 mg/kg) in BALB/c mice. At 24–96 h post-LPS mice had higher inflammatory cell counts in their BAL compared to sham and baseline mice (A; N = 4–9). At 48–96 h post instillation, LPS exposed mice also displayed increased BAL protein content compared to sham and baseline (B; N = 3–23). No differences were observed between LPS and sham mice across either of these outcomes at baseline, 4-h, or 240-h time point. At 48 h and 96 h post instillation, LPS exposed mice displayed decreased dynamic compliance (C; N = 3–20) compared to baseline and sham. At 48 h post saline instillation mice also displayed lower dynamic compliance compared to baseline. At 48 h but not 240 h post instillation, LPS exposure increased total lung histology lung injury score compared to baseline and sham mice (D; N = 3). Each data point represents the mean of an individual animal, with error bars indicating standard error of the mean. ^†^*P* < 0.05 versus baseline (0 h) and ^∗^*P* < 0.05 versus Sham. [Statistical analysis: Two-way ANOVA + Sidak's multiple comparisons test (A–D)]. BAL = bronchoalveolar lavage; Crs = dynamic compliance; IT = intratracheal; LPS = lipopolysaccharide.

**Supplemental Figure S1:**
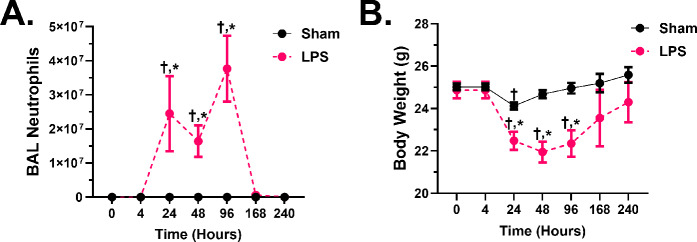
Pulmonary neutrophil recruitment and illness severity induced by intratracheal (IT) LPS (9 mg/kg) in BALB/c mice. Compared to baseline and sham mice, LPS instilled mice had increased BAL neutrophils (A) and lower body weights (B; 4-136) at 24 to 96 h post instillation (C; 4-136). No differences were observed between LPS and sham mice across any of these outcomes at baseline, 4-h, or 240-h time point. However, saline instillation resulted in lower body weights compared to baseline mice at 24 h post instillation. Each data point represents the mean of individual mice, with error bars indicating standard error of the mean. ^†^*P* < 0.05 vs baseline (0 h) and ^∗^*P* < 0.05 vs Sham. [Statistical analysis: Two-way ANOVA + Sidak's multiple comparisons test (A, B)]. BAL = bronchoalveolar lavage, IT = intratracheal, LPS = lipopolysaccharide.

Intratracheal instillation of LPS resulted in more total inflammatory cells (Fig. 1A) and elevated protein content (Fig. 1B) in the BAL compared to sham and baseline at 24, 48, and 96 h post instillation, returning to baseline levels at 168–240 h. At these times, BAL neutrophils were also elevated, and weight loss, as an alternative marker for illness severity, was found to be worse compared to sham and baseline (supplemental Fig. S1). At 24 h post instillation, sham mice also showed decreased body weight compared to baseline. However, by 168–240 h post instillation, no differences were observed between LPS and sham mice across any of these markers.

**Supplemental Figure S2:**
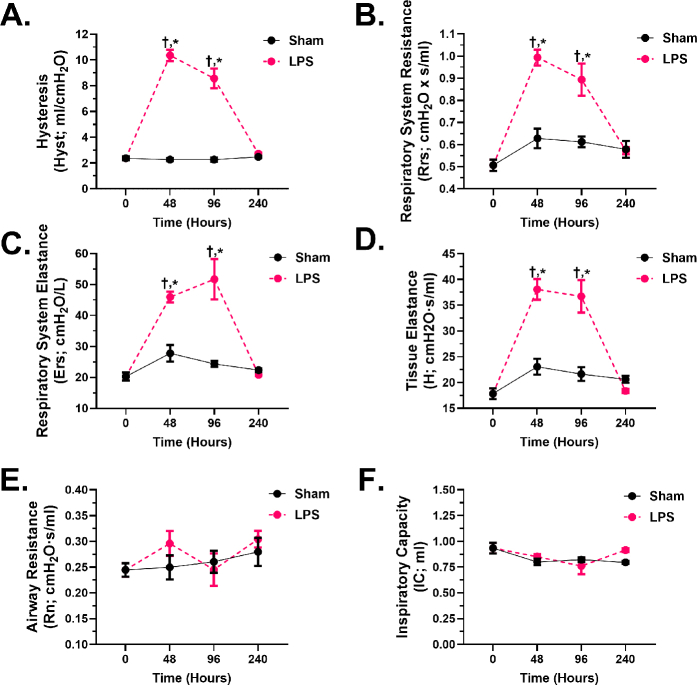
Impaired lung mechanics induced by intratracheal (IT) LPS (9 mg/kg) in BALB/c mice. At 48 h post instillation, LPS exposed mice displayed increased hysteresis (A; N = 3-20), respiratory system resistance (B; N = 3-20), respiratory system elastance (C; N = 3-20) and tissue elastance (D; N = 3-20) compared to sham and baseline. Saline instillation also increased these parameters at 48 and 96 h post instillation compared to baseline. No differences were observed between treatment groups across any of the time points for airway resistance (E; N = 3-20) or inspiratory capacity (F; N = 3-20). Each data point represents the mean of individual mice, with error bars indicating standard error of the mean. ^†^*P* < <0.05 vs baseline (0 h) and ^∗^*P* < 0.05 vs Sham. [Statistical analysis: Two-way ANOVA + Sidak's multiple comparisons test (A–F)]. IT = intratracheal, LPS = lipopolysaccharide, Hyst = hysteresis, Rrs = respiratory system resistance, Ers = respiratory system elastance, H = tissue elastance, Rn = airway resistance, IC = inspiratory capacity.

At the 48- and 96-h timepoints, mice instilled with LPS had worse dynamic compliance (Fig. 1C) compared to sham and baseline. Sham mice also had worse dynamic compliance compared to baseline at 48 h post instillation. In addition, LPS exposed mice displayed increased hysteresis, respiratory system resistance, respiratory system elastance, and tissue elastance compared to baseline and sham mice at these time points, with no differences across airway resistance or inspiratory capacity (supplemental Fig. S1). No differences between treatment groups or compared to baseline were observed for measurements of lung mechanics by 240 h post instillation. LPS exposed mice also displayed higher total lung injury histology scores at 48 h post instillation (Fig. 1D; Representative images provided in supplemental Fig. S2) compared to sham and baseline. Individual parameters of the injury score (supplemental Table S1) identified increased parenchymal inflammation, septal thickening, and edema, but not RBCs in the alveolar space. By 240 h post instillation, no differences were observed between LPS exposed mice and sham across total lung injury score or any of the individual parameters.

### Surfactant endpoints: Recovery, dysfunction, and composition

**Fig. 2:**
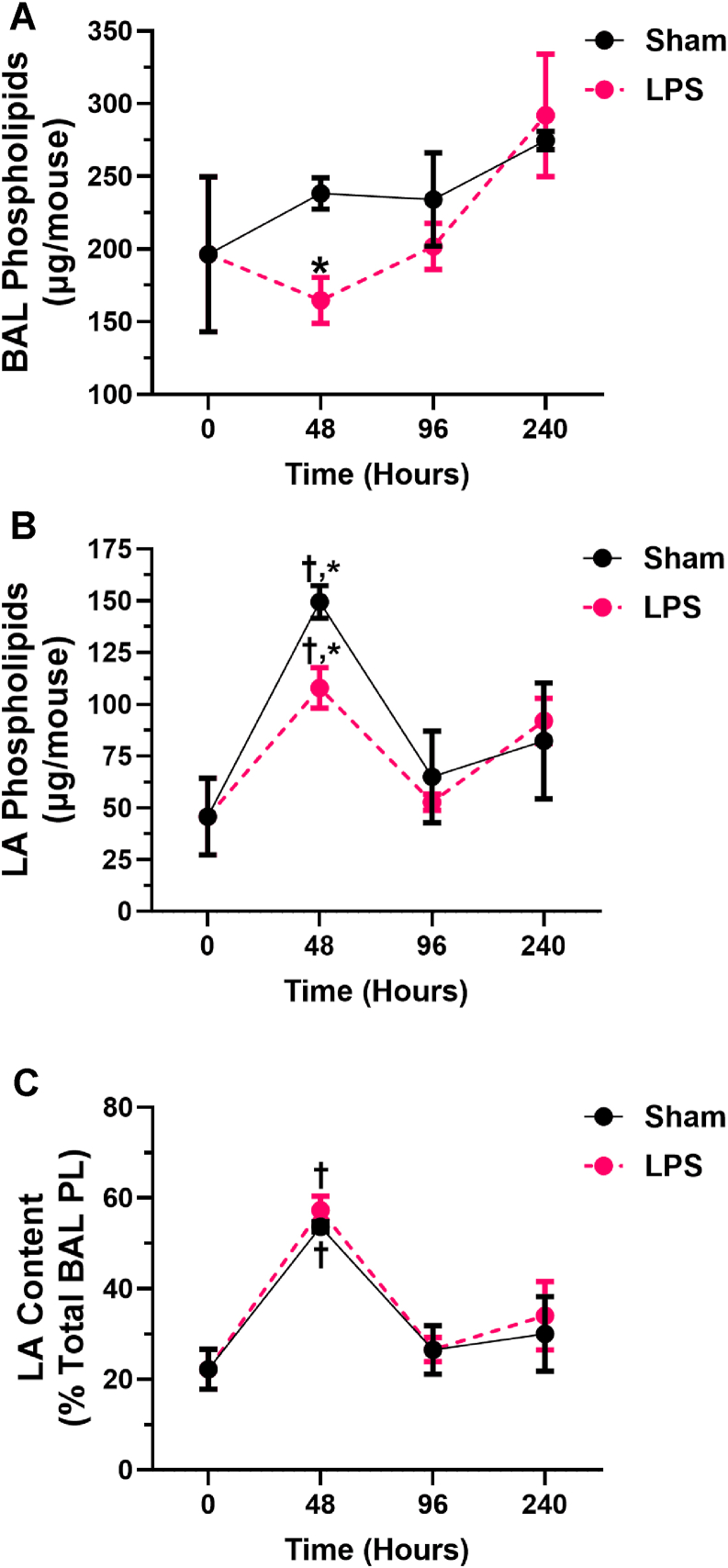
Surfactant deficiency induced by IT LPS (9 mg/kg) in BALB/c mice is evident at 48 h but resolved by 96–240 h. Compared to sham, LPS-instilled mice had reduced phospholipid content in the BAL (A; N = 3–21) and LA surfactant subfraction (B; N = 3–21) at the 48-h time point. No differences were observed between treatment groups at any other time points. Compared to baseline, saline and LPS instillation resulted in higher LA phospholipids recovered at 48 h post instillation. Further, no differences in percent LA were observed between LPS or sham mice (C; N = 3–21). However, at the 48-h time point, both saline and LPS instillation resulted in a higher percent LA compared to baseline. Each data point represents the mean of pooled LA, with error bars indicating the standard error of the mean. ^†^*P* < 0.05 versus baseline (0 h) and ^∗^*P* < 0.05 versus Sham. [Statistical analysis: Two-way ANOVA + Sidak's multiple comparisons test (A–C)]. IT, intratracheal; LA, large aggregates; LPS, lipopolysaccharides; PL, phospholipids.

In response to intratracheal LPS, total surfactant recovered in the BAL (Fig. 2A) and LA subfraction (Fig. 2B) were decreased at 48 h but did not differ compared to sham at any other timepoints. The data in these figures also demonstrated an increase in recovery of LA phospholipids in the sham exposed animals at 48 h compared to baseline, with a corresponding numerical increase in whole lavage phospholipids. The recovery of phospholipids at 48 h post intratracheal LPS also demonstrated an increase in the LA pellet, but no change in the total lavage phospholipids. Since the fraction of phospholipids within the LA pellet generally reflects phospholipids recently secreted by ATII cells, the relative distribution of total lavage phospholipids within the LA surfactant subfraction was analyzed. Although no differences were observed between treatment groups at any time points measured (Fig. 2C), both instillation of LPS and saline increased the percentage LA relative to whole lavage phospholipids at 48 h compared to baseline. Over the remainder of the time course post instillation (96 and 240 h) no differences were observed between baseline (untreated) and sham- or LPS-exposed animals for phospholipids in the BAL or LA.

**Fig. 3:**
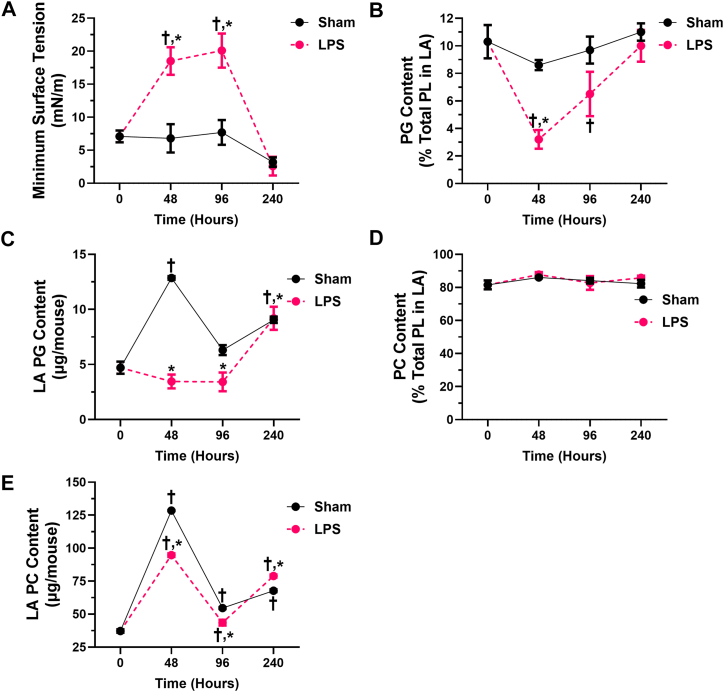
Surfactant dysfunction and PG depletion induced by IT LPS (9 mg/kg) in BALB/c mice is evident at 48 h but resolved by 240 h. LA isolated from sham and baseline mice achieved lower minimum surface compared to LPS exposed at 48 and 96 h post instillation, with no differences observed at the 240-h time point (A; N = 3–7). Compared to sham mice, LPS expose mice also had lower proportion of LA PG at 48 h and numerically lower LA PG at 96 h, but not 240 h post instillation (B; N = 3–7). Compared to baseline, LPS instillation resulted in lower percentage of PG in the LA at both 48 and 96 h post instillation. Similarly, the amount of LA PG was lower at 48 h and 96 h, but not 240 h post instillation of LPS compared to sham (C; N = 3–7). Compared to baseline, saline instillation also resulted in a higher amount of PG recovered at 48 and 240, but not 96 h post instillation. Although no differences were observed in the proportion of PC in LA at any peak injury or recovery time points (D; N = 3–7), the amount of LA PC was lower in LPS instilled mice at 48 and 96 h post instillation compared to sham, yet higher at 240 h (E; N = 7). In addition, LPS and saline instillation resulted in more PC recovered at 48, 96, and 240 h post instillation compared to baseline. Each data point represents the mean of pooled LA, with error bars indicating standard error of the mean. ^†^*P* < 0.05 versus baseline (0 h) and ^∗^*P* < 0.05 versus Sham. [Statistical analysis: Two-way ANOVA + Sidak's multiple comparisons test (A–E)]. IT, intratracheal; LA, large aggregates; LPS, lipopolysaccharides; PC, phosphatidylcholine; PG, phosphatidylglycerol; PL, phospholipids.

Surfactant dysfunction, demonstrated by increased values for minimum surface tension in the LA pellets isolated from LPS-exposed mice compared to sham mice occurred at 48 and 96 h (Fig. 3A). Surface tension-lowering activity post LPS normalized and was not different compared to sham animals at 240 h post instillation. Similarly, at a set lipid concentration, analysis of surfactant phospholipid composition revealed the relative proportion of PG in the LA pellet to be reduced in mice exposed to LPS at 48 h as compared to sham mice (Fig. 3B). In addition, absolute recovery of PG in the LA pellet was numerically lower (4.70 μg/mouse baseline vs. 2.96 μg/mouse LPS) at 48 h post intratracheal LPS compared to mice at baseline. The decrease in PG at 48 h post LPS occured despite the increase in absolute PG at 48 h post saline instillation relative to baseline, which remained higher at 240 h (Fig. 3C). No differences in PG (relative or absolute) were observed after ALI resolution at 240 h post instillation.

**Supplemental Figure S4:**
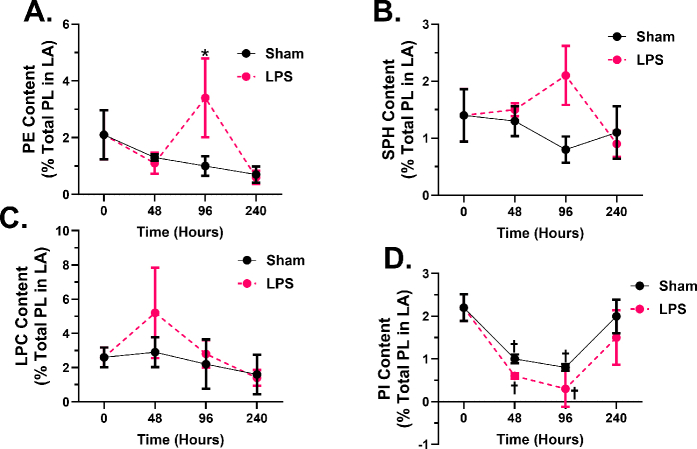
IT LPS (9 mg/kg) in BALB/c mice does not alter the relative proportion of major surfactant phospholipids in the LA surfactant subfraction. No differences were observed at peak injury or recovery time points with respect to the percentage of PE (A; N = 3-7), SPH (B; N = 3-7), LPC (C; N = 3-7), or PI (D; N = 3-7) comprising the LA surfactant subfraction. However, compared to saline both saline and LPS instillation resulted in higher relative PI compared to baseline at 48 and 96 h post instillation. Each data point represents the mean of pooled samples, with error bars indicating standard error of the mean. ^†^*P* < 0.05 vs baseline (0 h) and ^∗^*P* < <0.05 vs Sham. [Statistical analysis: Two-way ANOVA + Sidak's multiple comparisons test (A–D)]. IT = intratracheal, LPS = lipopolysaccharides, PL = phospholipids, LA = large aggregates, PE = phosphatidylethanolamine, SPH = sphingomyelin, LPC = lysophosphatidylcholine, PI = phosphatidylinositol.

**Supplemental Figure S5:**
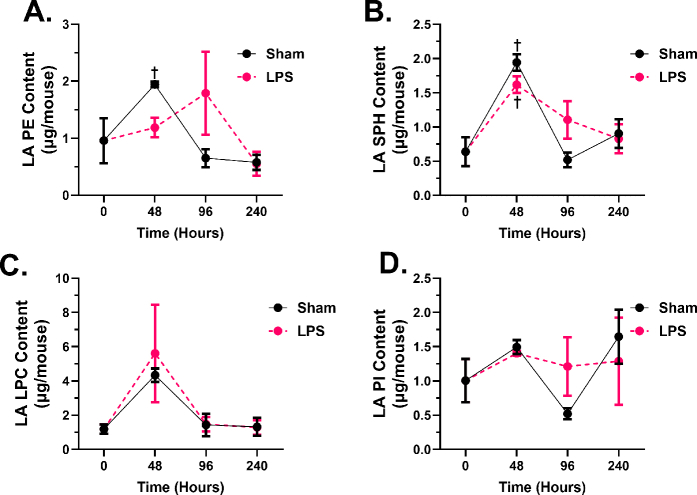
IT LPS (9 mg/kg) in BALB/c mice does not alter the absolute amount of major surfactant phospholipids recovered in the LA. No differences were observed at peak injury or recovery time points with respect to the amount of LA PE (A; N = 3-7). SPH (B; N = 3-7), LPC (C; N = 3-7), or PI (D; N = 3-7). However, compared to baseline, saline instillation increased the amount of LA PE recovered at 48 h post instillation. Both saline and LPS instillation also resulted in more LA SPH recovered at 48 h compared to baseline. Each data point represents the mean of pooled samples, with error bars indicating standard error of the mean. ^†^*P* < 0.05 vs baseline (0 h) and ^∗^*P* < 0.05 vs Sham. [Statistical analysis: Two-way ANOVA + Sidak's multiple comparisons test (A-D)]. IT = intratracheal, LPS = lipopolysaccharides, PL = phospholipids, LA = large aggregates, PE = phosphatidylethanolamine, SPH = sphingomyelin, LPC = lysophosphatidylcholine, PI = phosphatidylinositol.

There were no differences observed in PC, the most abundant surfactant phospholipid, when analyzed as the relative proportion (Fig. 3D) utilized for the measurements of surfactant function. However, the absolute amount of PC in the LA pellet post intratracheal LPS instillation was increased as compared to baseline prior to instillation (Fig. 3E). This increase in PC was lower than the increase observed after the sham (saline) instillation and remained decreased compared to sham at 96 h. Absolute levels of PC recovered in the LA pellet at 240 h were also increased in comparison to both sham controls and baseline (Fig. 3E). Between treatment groups or compared to baseline, no differences were observed across relative proportion (supplemental Fig. S4) or absolute amount (supplemental Fig. S5) recovered for other individual surfactant phospholipids measured including either of the 2 lysophospholipids analyzed (LPC and LPG). Data for LPG were not shown since the results from the LA pellet in all samples were below the detection limits in our HPLC system.

### Phospholipase activity

**Fig. 4:**
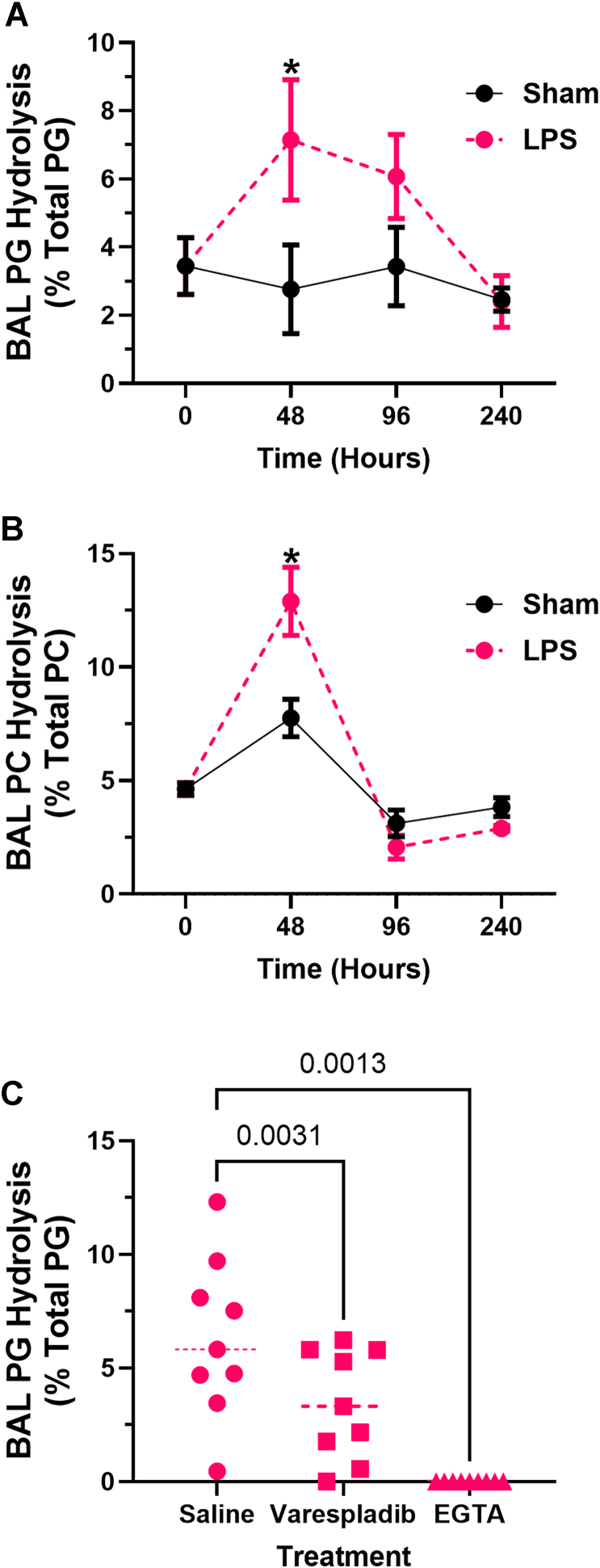
Alveolar sPLA_2_-mediated PG and PC hydrolysis induced by IT LPS (9 mg/kg) in BALB/c mice is evident at 48 h, but not 240 h post instillation, and is reduced ex vivo by an sPLA_2_ inhibition or calcium chelating agent. Compared to sham mice, BAL from LPS exposed mice had increased sPLA_2_ activity at 48-h for both PG (A; N = 9–17) and PC (B; N = 3–7) phospholipids compared to sham, with no differences observed by 240 h. Although PC hydrolysis was not different at the 96-h time point, PG hydrolysis was still numerically elevated compared to sham. Compared to saline treatment, both Varespladib (50 μm) and EGTA (5 mM) reduced PG hydrolysis for BAL isolated from LPS exposed mice at 48 h post intratracheal instillation (C; N = 9). Each data point represents the mean of pooled samples (A and B) or individual mice (C). Error bars indicate the standard error of the mean (A and B), while the horizontal line indicates mean of individual mice (C). ^†^*P* < 0.05 versus baseline (0 h) and ^∗^*P* < 0.05 versus Sham. [Statistical analysis: Two-way ANOVA + Sidak’s multiple comparisons test (A and B); Repeated One-way ANOVA + Dunnett’s multiple comparisons test (C)]. BAL, bronchoalveolar lavage; EGTA, Ethylene glycol-bis(β-aminoethyl ether)-N,N,N′,N′-tetraacetic acid; IT, intratracheal; LA, large aggregates; LPS, lipopolysaccharides; PC, phosphatidylcholine; PG, phosphatidylglycerol; PL, phospholipids; sPLA_2_, secretory phospholipase A2.

Compared to sham, BAL from LPS-exposed mice had increased sPLA_2_-mediated PG (Fig. 4A) and PC (Fig. 4B) hydrolysis at the 48-h time point, when assayed ex vivo against surfactant vesicles. Although PG hydrolysis was also numerically increased at the 96-h timepoint in LPS compared to sham mice, no differences were observed for PC. Further, no differences in BAL PG or PC hydrolysis were observed at 240 h. At the 48-h time point, treatment of BAL from LPS-instilled mice with either the sPLA_2_ inhibitor, Varespladib or the calcium chelating agent, EGTA (Fig. 4C) reduced sPLA_2_-mediated PG hydrolysis compared to saline treatment.

### Alterations in surfactant proteins

**Fig. 5:**
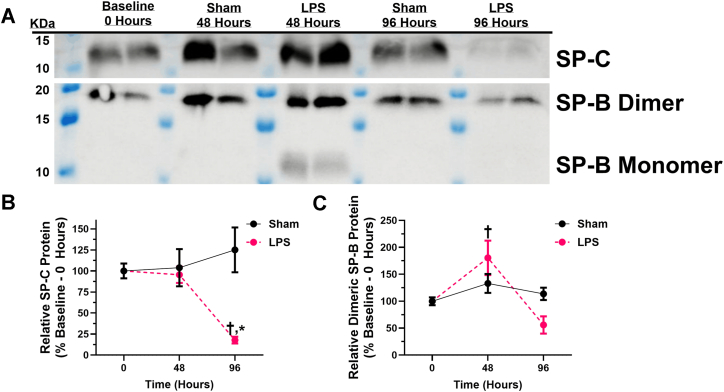
Hydrophobic pulmonary surfactant protein depletion induced by IT LPS (9 mg/kg) in BALB/c mice is evident at 96 h. Representative Western blot images of SP-C as well as the SP-B dimer and monomer (A; N = 8–9). Compared to sham and baseline, BAL isolated from LPS exposed mice had lower SP-C (B) and numerically lower dimeric SP-B (C; N = 6) protein expression at 96 h post instillation. Although LPS instillation resulted in higher SP-B expression at 48 h post instillation compared to baseline, no differences were observed between LPS and sham mice across either surfactant protein. The abnormal appearance of the SP-B monomer only occurred in LPS-treated mice at the 48-h time point. Each data point represents an individual animal, with horizontal lines indicating mean. ^†^*P* < 0.05 versus baseline (0 h) and ^∗^*P* < 0.05 versus Sham. [Statistical analysis: Two-way ANOVA + multiple comparisons test (B and C)]. IT, intratracheal; LPS, lipopolysaccharide; SP-B, surfactant protein B; SP-C, surfactant protein C.

At 96 h post instillation, LPS-exposed mice displayed lower protein levels for BAL SP-C compared to sham animals, while levels of SP-C did not differ between groups at the 48-h time point (Fig. 5B). Compared to sham, LPS-exposed mice also had numerically lower BAL SP-B protein at 96 h, which did not differ at 48 h (Fig. 5C). In contrast, comparison of surfactant protein levels between 48-h and 0-h time points revealed numerically higher levels of SP-B in both the sham and LPS-exposed mice. Levels of SP-C at 48 h did not differ in comparison to the 0-h time point. Uniquely, a combination of dimeric and monomeric forms of SP-B were observed in the LPS-exposed mice at 48 h, while only the dimeric form of SP-B was detected under all other conditions analyzed.

### Treatment with varespladib at peak ALI

**Fig. 6:**
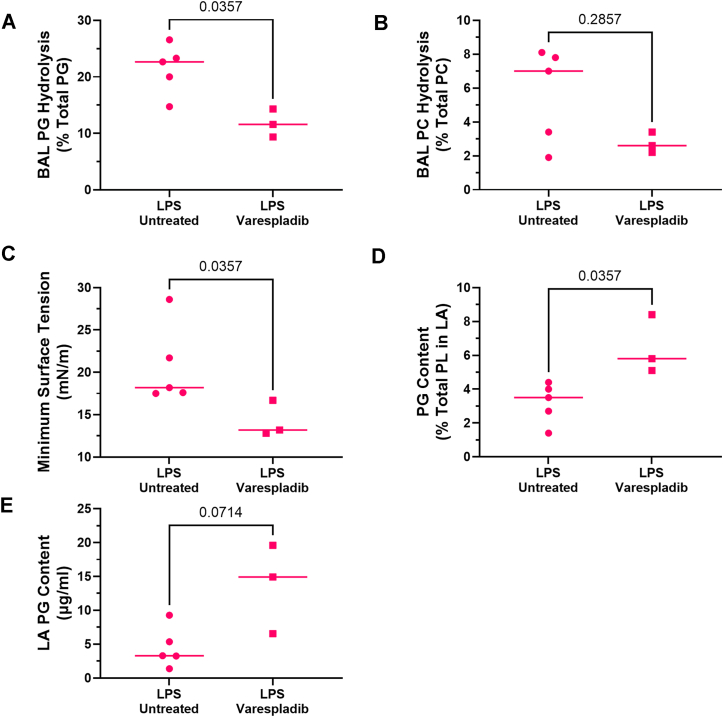
Varespladib inhibits pulmonary sPLA_2_ activity and restores surfactant function, as well as PG content in mice treated 48 h after IT LPS (9 mg/kg). At 4-h post treatment, BAL isolated from mice instilled with LPS and treated with IT varespladib (5.7 mg/mouse) had reduced BAL sPLA_2_-mediated PG hydrolysis (A; N = 3–5), and numerically reduced sPLA_2_-mediated PC hydrolysis compared to untreated LPS controls (B; N = 3–5). LA isolated from these mice also achieved lower minimum surface tensions compared to untreated LPS controls (C; N = 3–5). In addition, varespladib treatment increased the relative proportion of PG (D; N = 3–5) and numerically increased the absolute amount of PG in LA (E; N = 3–5) in LPS instilled mice compared to untreated LPS controls. Each data point represents pooled samples of mice (A–D). Horizontal line indicates median. [Statistical analysis: Mann-Whitney test (A–D)]. BAL, bronchoalveolar lavage; PG, phosphatidylglycerol; PC, phosphatidylcholine; sPLA_2_, secretory phospholipase A2; LPS, lipopolysaccharides; PL, phospholipids.

**Supplemental Figure S6:**
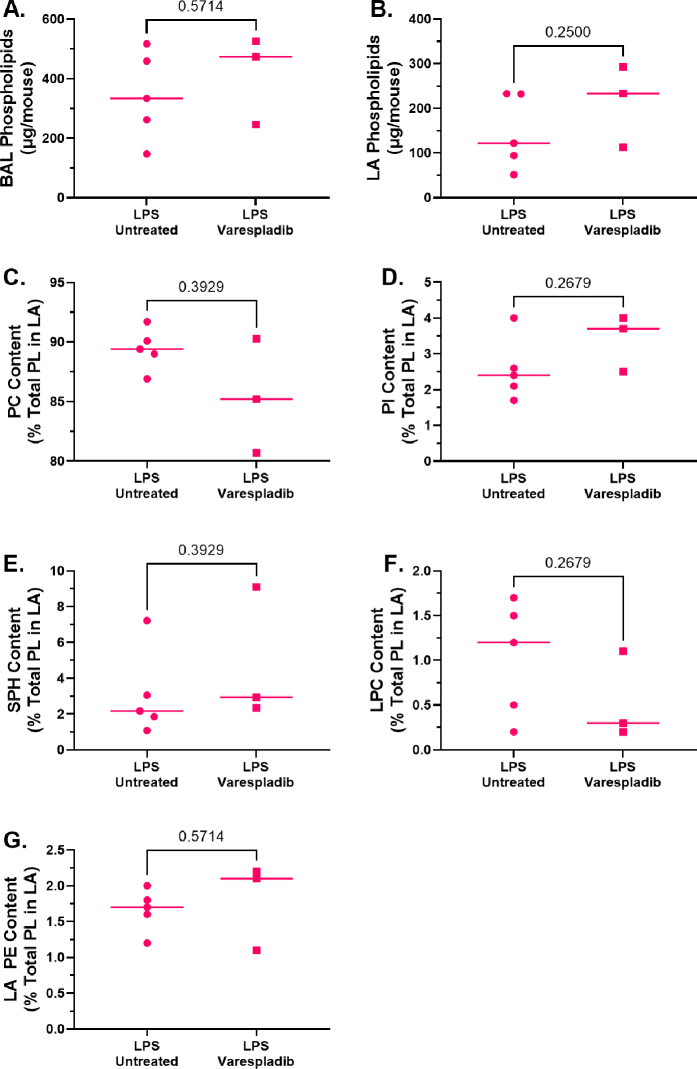
Varespladib treatment of mice after a 48-h LPS insult did not increase the amount of surfactant recovered nor the relative proportion of major surfactant phospholipids in the LA surfactant subfraction. No differences were observed between varespladib and untreated LPS mice in BAL (A; N = 3-5) or LA (B; N = 3-5) phospholipids. At 4-h post treatment, LA isolated from Varespladib and untreated LPS mice also had similar percentages of PC (C; N = 3-5), PI (D; N = 3-5), SPH (E; N = 3-5), LPC (F; N = 3-5), and PE (G; N = 3-5). Each data point represents pooled animals, while the horizontal line indicates median. [Statistical analysis: Mann-Whitney test (A–L)]. IT = intratracheal, LPS = lipopolysaccharides, LA = large aggregates, PC = phosphatidylcholine, PI = phosphatidylinositol, SPH = sphingomyelin, LPC = lysophosphatidylcholine.

Treatment of mice at the peak of ALI (48 h post LPS) with intratracheal varespladib resulted in reduced PG hydrolysis activity in the BAL supernatant compared to untreated LPS controls at 52 h post LPS (Fig. 6A). Numerical reductions in PC hydrolysis were also observed following varespladib treatment (Fig. 6B). The function of the LA pellets isolated from LPS instilled mice after treatment with varespladib was improved as demonstrated by reduced minimum surface tensions compared to untreated LPS controls (Fig. 6C). Compared to untreated LPS controls, reductions in PG hydrolysis following intratracheal varespladib was also associated with an increase in the relative percentage of PG in the LA pellet (Fig. 6D) used at normalized phospholipid concentrations during PBS measurements of surface activity, and total PG recovered within the LA pellet (Fig. 6E). No differences in the relative percentage or total content within the LA were observed across any of the other major phospholipid groups measured, including PC (supplemental Fig. S6). Compared to untreated LPS controls, the recovery of surfactant in the BAL fluid and LA phospholipids were not altered after varespladib treatment.

## Discussion

In the current study, we examined a transient, non-lethal murine model of LPS-induced ALI to explore whether a synchronized in vivo relationship between surfactant dysfunction, PG depletion, and sPLA_2_ activity was present at peak ALI. Although suggested from studies of multiple investigators including our prior in vitro studies and analysis of BAL fluid from patients with ARDS (21, 35, 41, 42, 47, 57), no pre-clinical model has previously affirmed the synchronous presence of all. As a first step, we confirmed that our lab’s application of the intratracheal LPS model included the essential features of ARDS, as recommended by prior consensus definitions for ALI models (43). Intratracheal LPS produced a time-dependent pattern of (*1*) lung inflammation (immune cell influx), (*2*) alveolar–capillary barrier permeability (protein leak), (*3*) lung injury (histology), and (*4*) physiologic dysfunction (impaired respiratory mechanics). Furthermore, the murine model displayed clear progression to, and recovery from ALI (at 240 h), with peak changes occurring between 48 to 96 h. Having established the time course for peak ALI, we focused on the specific surfactant endpoints of primary interest. Notably, surfactant dysfunction was present at peak ALI time points of 48 and 96 h and was associated with both increased alveolar sPLA_2_ activity and decreased PG content in the LA pellet, the active surfactant subfraction. In contrast, periods of resolution from LPS-induced ALI were not associated with altered surfactant function, PG content, or sPLA_2_ activity. These observational findings were further supported by our results using ex vivo and in vivo interventional experiments with varespladib, a general inhibitor of sPLA_2_ activity. During peak ALI, treatment with intratracheal varespladib reduced alveolar sPLA_2_ activity, improved surfactant function, and increased PG content. The combined results were consistent with and provide new, supportive in vivo evidence for our hypothesis that increased sPLA_2_ activity and depletion of PG contribute to the surfactant dysfunction of ALI.

Surfactant dysfunction was only observed at times of peak LPS-induced ALI and was associated with impaired lung mechanics. HPLC examination of the major phospholipid groups within surfactant at the 48-h timepoint, revealed only a reduction in the amount of PG for LPS exposed animals compared to sham controls. Notably, levels of PG post intratracheal LPS were reduced when quantified as both total amounts recovered in the surface-active LA pellet within the BAL, and the relative proportion of total LA phospholipids. The latter of which being more directly related to the surface activity measurements made using the PBS, since those measurements and comparisons were performed at a normalized, fixed lipid concentration of 1 mg/ml. By comparison, total levels of PC recovered in the LA pellet were increased at 48 h post intratracheal LPS but did not differ when quantified as the relative amount of LA phospholipids used for surface activity analyses. Similar to PC, no differences were seen in the relative amounts of all other major phospholipid groups including PE, LPC, LPG, and SPH at 48 h post intratracheal LPS in the LA used for surface activity measurements. The absence of change in PI was also noteworthy since PI represents the second most abundant anionic phospholipid. Some have speculated that PI might potentially support the critical interaction with SP-B, as an alternative to PG (58). In ARDS patients, small increases in PI have been shown, but its ability to fully substitute PG’s functional interaction with SP-B has not been studied. Lastly, the correspondence between surfactant dysfunction and an isolated reduction in PG was seen at 48 h post intratracheal LPS despite no reduction in levels of either hydrophobic surfactant protein, SP-B or SP-C, within the LA pellet.

When quantified as absolute amounts recovered within the lavage fluid including total lavage or isolated within the LA pellet, the interpretation must include consideration of the dynamic nature of alveolar surfactant levels, and potential effects of the instillation procedures. From that viewpoint, our results demonstrated an increase in recovery of LA phospholipids in the sham exposed animals at 48 h compared to baseline without a corresponding change in whole lavage phospholipids. These changes most likely reflect secretion of new surfactant by alveolar type II (ATII) cells in response to the instillation procedure. The recovery of phospholipids at 48 h post intratracheal LPS also demonstrated no change in the total lavage, with an increase in the LA pellet compared to baseline. Since the fraction of phospholipids within the LA pellet generally reflects phospholipids recently secreted by ATII cells, these combined changes suggest a similar secretion post the intratracheal LPS instillation likely occurred. The numerical increases in SP-B levels in the LA pellets at 48 h (sham and LPS exposed) may be further evidence for increased new surfactant secretion as a compensatory mechanism in response to the installation and inflammatory damage, as described in other forms of lung injury including meconium aspiration (58). Over the remainder of the time course post instillation (96 and 240 h) no differences were observed between baseline (untreated) and sham or LPS exposed animals for phospholipids in the BAL or LA.

Extension of the analysis of the absolute recovery of phospholipids in the lavage fluid to individual phospholipid groups reveals that LA PG was the only major phospholipid species reduced at 48 h post-intratracheal LPS compared to sham mice. This reduction in the absolute recovery of PG in the LA pellet persists at 96 h post-LPS but was not different at 240 h. It was also notable that the decrease in PG at 48 h post LPS occurred despite the increase in PG at 48 h post saline instillation compared to baseline, which remained numerically higher at 240 h. Similarly, at 48 and 96 h post LPS instillation, the absolute levels of PC were also lower than the sham control group. However, compared to baseline, PC levels were higher in both the LPS and sham groups at 48 h, and remained increased over the entire experimental time course post instillation of saline or LPS. Among the other major phospholipid groups, increases in recovery of all groups compared to baseline, except PI, were seen at 48 h post saline instillation, consistent with the increase in the LA pellet. No differences in their recovery were seen at either 96 or 240 h. At 48 h post LPS exposure, the only other change in relative PL recovery (beyond PG and PC) was an increase in SPH compared to sham, which was not present at either 96 or 240 h. Taken together, analysis of the changes in absolute PL levels recovered from the lung lavage further highlight a disproportionate decrease in PG, most notably at 48 h after LPS instillation.

Our studies also demonstrate increased BAL sPLA_2_-mediated phospholipid hydrolysis activity for PG- and PC-labeled substrates that peak and resolve over the same time course of ALI post intratracheal LPS. Inhibition of hydrolysis by a calcium chelator (EGTA) was consistent with the millimolar-calcium dependence of an sPLA_2_ and argues against calcium-independent PLA_2_ enzymes, such as the intracellular PLA_2_ and lysosomal PLA_2_ (32, 33). Likewise, ex vivo inhibition of BAL sPLA_2_ activity by varespladib, a well-characterized inhibitor for sPLA_2_ isoforms IIA, V, and X (59, 60, 61), makes the calcium-dependent cytosolic PLA_2_ an unlikely contributor. Progression of those studies to an in vivo model with intratracheal instillation of varespladib to mice at peak ALI (48 h post LPS) similarly demonstrated a reduction in sPLA_2_ hydrolysis activity at 4 h post instillation. In addition, the reduced hydrolysis of PG was associated with increased PG content and improved surfactant function. Although not definitive, these initial experiments targeting sPLA_2_-mediated hydrolysis as a specific mechanism for the PG depletion caused by intratracheal LPS, and their potential role in surfactant dysfunction in ALI, are further supportive and encouraging.

The phospholipase studies completed thus far, only examined the localized sPLA_2_ activity with the alveolar lavage fluid compartment. Notably, the time course of increased alveolar sPLA_2_ activity closely parallelled the development and resolution of lung inflammation, with increased enzymatic activity coinciding primarily during periods of cellular infiltration, predominantly neutrophils, into the alveolar space. Although neutrophils are a well-established source of multiple sPLA_2_ isoforms including group IIA, V, and X (27, 28, 33), our studies did not explore or exclude the multiple other potential sources of sPLA_2_ that may be contributing from within the alveolar environment (alveolar macrophages and epithelium) or through serum leak of sPLA_2_ activity that was released by other organs. Similarly, the in vivo studies using intratracheal instillation of varespladib most likely reflected an impact on active enzyme secreted within the alveolar fluid compartment, but do not exclude other direct effects on sPLA_2_ activity from alveolar cells or serum. The positive impact of sPLA_2_ inhibition on surfactant function in both ex vivo and in vivo studies naturally raises questions regarding which of the multiple human sPLA_2_ subtypes are responsible. The sPLA_2_-IIA is an attractive candidate based on its established expression in the human lung, upregulation in inflammatory conditions, and reported preference for the anionic phospholipid PG (28, 32, 41). Other studies utilizing sPLA_2_-V knockout mice demonstrate resistance to ALI from intratracheal LPS but did not examine changes in surfactant function or composition (32, 35, 62). Based on multiple prior studies demonstrating increased sPLA_2_ activity in a variety of inflammatory lung conditions, the observed BAL hydrolysis in ALI induced by intratracheal LPS likely reflects the combined effects of more than a single sPLA_2_ isoform (63, 64).

Importantly, the absence of substantial accumulation of lysophospholipids in our model provides additional mechanistic insight into the relationship between sPLA_2_ activity and surfactant dysfunction. Despite clear evidence of increased sPLA_2_-mediated phospholipid hydrolysis, we did not observe significant elevations in lysophospholipid species, including LPC and LPG, within the surface-active LA fraction. At peak ALI, instillation of LPS only increased LPC levels by 1.5%–2% (total LA phospholipids) compared to baseline, which was not different from levels observed in saline instilled controls. This finding is consistent with the rapid metabolism and cellular uptake of lysophospholipids in vivo, where these intermediates are efficiently re-acylated or cleared by alveolar cells, limiting their accumulation in the extracellular compartment (65, 66). While prior studies have demonstrated that lysophospholipids can impair surfactant function, these effects typically require substantially higher concentrations, 10%–15% of LA phospholipid (41, 42), than those observed in the current study. Thus, the lack of significant lysophospholipid accumulation argues against a primary role for these hydrolytic byproducts in mediating surfactant dysfunction in our model. Instead, our findings further support the concept that depletion of PG itself, rather than accumulation of lysophospholipids, is a more significant contributor to impaired surfactant function during LPS-induced ALI.

The persistence of surfactant dysfunction at 96 h was seen even as PG content in the LA pellet and sPLA_2_-mediated PG hydrolysis in the BAL supernatant began to normalize, suggesting that the altered surfactant function was potentially less attributable to PG depletion or ongoing phospholipid hydrolysis. Notably, this persistent biophysical surfactant dysfunction coincided with significantly reduced BAL SP-C levels, and numerically lower levels of SP-B at 96 h. Given the important role of SP-C in stabilizing surfactant films, its deficiency at 96 h serves as a plausible contributing mechanism (10, 11). Further, the potential role of the combined effects from numerical changes in both SP-B and PG at 96 h cannot be excluded. In addition to the changes in surfactant proteins, our data also demonstrated persistently increased BAL protein at 96 h that were numerically higher (*P* = 0.1319) than at 48 h. Multiple plasma proteins that leak into the alveoli during ARDS have been demonstrated to disrupt or inhibit surfactant function including albumin, fibrinogen, and CRP (67, 68, 69). Of note, most BAL proteins leaking from plasma were separated by ultracentrifugation from the LA pellet used for functional surfactant measurements. However, the presence of inhibitory proteins that might co-migrate with LA lipids during ultracentrifugation cannot be excluded and may be contributing more to surfactant dysfunction at 96 h as compared to 48 h. In aggregate, the alterations in SP-C and SP-B provide further supportive evidence that inflammation-driven and temporally distinct alterations to the hydrophobic protein components of surfactant’s composition contribute to impaired surfactant function and the pathophysiology of ARDS.

Our results also highlight the temporal association between changes in PG content, surfactant dysfunction and impaired lung mechanics, which further enhances their potential clinical relevance (70, 71). As it relates to lung mechanics in ARDS, surfactant dysfunction would be most anticipated to correspond with reduced lung compliance and increased hysteresis (5, 72). These functional impairments were demonstrated in our model and were notably reversible post-ALI resolution. As seen in our histology findings, other aspects of alveolar injury were also likely contributing to these impaired mechanics. With the absence of histological lung injury at 240 h after LPS instillation, impaired lung mechanics in our model appeared to be more reflective of dynamic inflammation-mediated biochemical injury rather than irreversible structural damage. The recovery of lung function may simply reflect the replacement of impaired or structurally altered surfactant with new de novo surfactant synthesis. Future sub-studies examining the effects of exogenous surfactant replacement at the peak of ALI are warranted and may shed additional light on the relative contribution of dysfunctional surfactant. Subsequent studies that extend our in vivo experiments with intratracheal varespladib to examine its effects on surfactant dysfunction at 96 h post intratracheal LPS are also warranted and may provide further insight for the relative contributions of increased sPLA_2_ and PG depletion versus other contributing variables such as hydrophobic surfactant proteins and plasma proteins.

While this study provides novel, supportive evidence for the associations between PG depletion, sPLA_2_-mediated PG hydrolysis, and surfactant dysfunction, several limitations should be acknowledged. First, these findings do not definitively establish causal, mechanistic relationships between increased sPLA_2_-mediated PG hydrolysis, PG depletion, surfactant dysfunction, and altered lung mechanics. For example, other mechanisms for PG depletion have been reported in a murine influenza model of ALI. In that model, PG depletion was linked to reduced availability of an essential liponucleotide substrate, cytidine 5′-diphospho-choline, for PG biosynthesis (73). The substantially longer time course to ALI post influenza limits direct comparisons with the current LPS model, but the intratracheal LPS model should be sufficient to test the significance of that molecular pathway in future sub-studies. Second, total or partial inactivation of surfactant proteins because of oxidative and degradative environments may further contribute to surfactant impairment and respiratory dysfunction. Detailed analysis of structure-function determinants of the surfactant protein fraction extracted from different time points of our model should provide further clues on the role of the proteins to amplify and later restore alveolar homeostasis. Third, more detailed characterization of alterations in major phospholipid classes, including changes in specific fatty acid side chains, presence of oxidized lipid species, and increased neutral lipids, particularly cholesterol, have been demonstrated in other studies (6, 22, 74, 75, 76). Contributions from these additional phospholipid modifications to the surfactant dysfunction observed in our model cannot be excluded and warrant further investigation using complementary analytical approaches on post intratracheal LPS samples. Fourth, our data do not directly confirm the presence or absence of specific sPLA_2_ isoform(s) responsible for the observed PG hydrolysis. Although our findings were consistent with a role for sPLA_2_-mediated hydrolysis, pulmonary levels of individual isoforms were not directly measured. Notably, in vivo and ex vivo studies using varespladib an inhibitor of sPLA2-IIA, V, and X, attenuated PG hydrolysis and associated changes in surfactant, suggesting that one or more of these isoforms likely contribute to the observed phenotype. However, definitive identification of the responsible isoform(s) will require future studies employing isoform-specific assays and transgenic knockout models. Fifth, our data do not examine potential and likely alterations in surfactant synthesis or recycling within ATII cells and alveolar macrophages caused by intratracheal LPS. These changes are very likely to be present, contribute to the overall surfactant findings we observed, and warrant further sub-studies. However, we would not expect them to be positively impacted by in vivo intratracheal varespladib over a 4-h time window and therefore are unlikely mechanisms for the improved surfactant function observed in those experiments. Finally, while including all the defining features of human ARDS, this non-lethal model represents a relatively mild form of ALI and does not capture the full severity or clinical consequences that can be seen in ARDS. Future studies including more translationally relevant ARDS triggers (e.g. respiratory infections, systemic sepsis) and larger animal species are needed to further validate and refine these findings. Nonetheless, the transient nature of the intratracheal LPS model enabled the study of both injury and recovery phases, providing mechanistic insights that would have been difficult to obtain in more severe or lethal models. Our results do indicate that future studies in models that include more severe forms of ALI and larger animals are warranted and will help inform and guide the design of those studies.

In summary, this novel in vivo study demonstrates a synchronous temporal relationship between surfactant dysfunction, depletion of PG, and increased sPLA_2_ activity including PG hydrolysis at the peak of LPS-induced ALI. These changes occur concurrently with pathophysiological lung impairment and resolve in parallel during recovery, supporting a dynamic and reversible process. In addition, ex vivo and in vivo inhibition of sPLA_2_ with varespladib attenuated PG hydrolysis, restored PG content, and improved surfactant function, providing functional evidence that links enzymatic activity to compositional and biophysical abnormalities in surfactant. Together, these findings extend prior in vitro and clinical observations suggestive of the potentially important contributions of PG and sPLA_2_ to surfactant dysfunction in ALI. Future sub-studies to more fully define the causative relationships of these pathways are warranted, and feasible within the non-lethal intratracheal LPS model.

## Data availability

All data supporting the findings of this study are contained within the manuscript and its supplementary materials. Any additional information or materials will be made available by the corresponding author upon reasonable request.

## Supplemental data

This article contains supplemental data.

## Conflict of interest

The authors declare that they have no conflicts of interest with the contents of this article.

## Figures and Tables

**Supplemental Figure S3:**
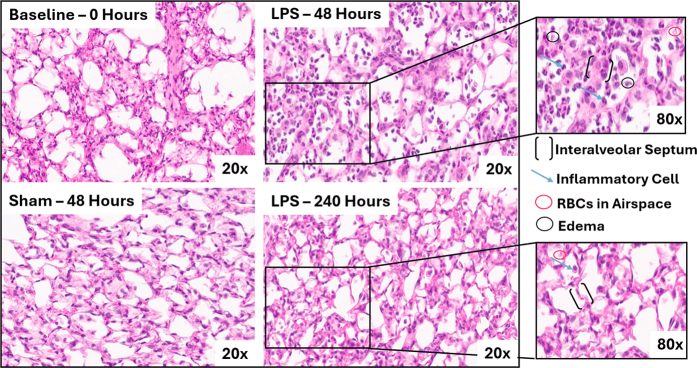
Representative whole lung sections in BALB/c mice used for histology scoring. Lungs were stained with H&E and images of the entire section were taken at 20x using a slide scanner. Black brackets indicate regions of interalveolar septal thickening, blue arrows indicate inflammatory cells, red circles indicate RBCs in the airspace, and black circles indicate edema. RBC = red blood cells.

**Supplemental Figure S7:**
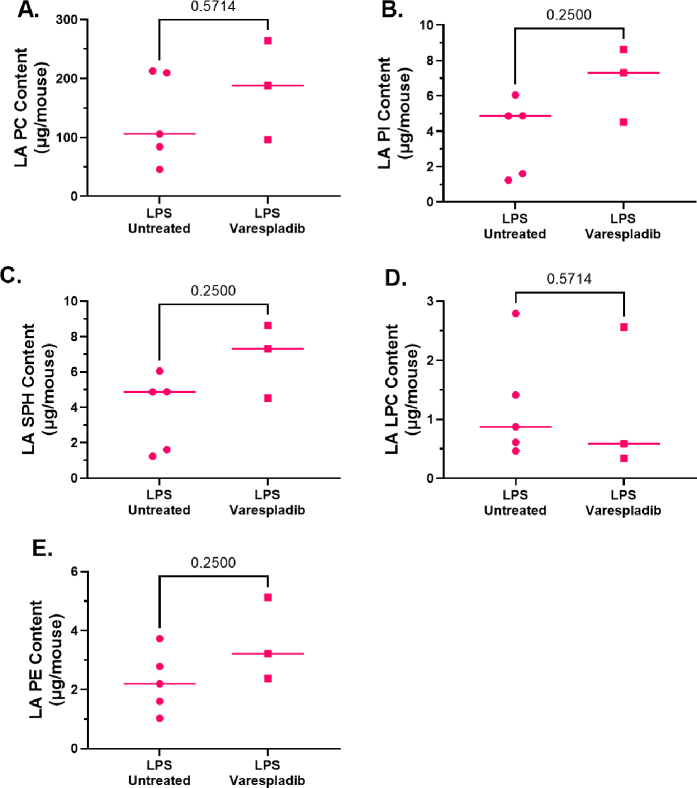
Varespladib treatment of mice after a 48-h LPS insult did not increase the absolute amount of major surfactant phospholipids in the LA. At 4-h post treatment, LA isolated from Varespladib and untreated LPS mice has similar absolute amounts of PC (A; N = 3-5) PI (B; N = 3-5), SPH (C; N = 3-5), LPC (D; N = 3-5), and PE (E; N = 3-5). Each data point represents pooled animals, while the horizontal line indicates median. [Statistical analysis: Mann-Whitney test (A–L)]. IT = intratracheal, LPS = lipopolysaccharides, LA = large aggregates, PC = phosphatidylcholine, PI = phosphatidylinositol, SPH = sphingomyelin, LPC = lysophosphatidylcholine.

## References

[1] Matthay M.A., Arabi Y., Arroliga A.C., Bernard G., Bersten A.D., Brochard L.J. (2024). A new global definition of acute respiratory distress syndrome. Am. J. Respir. Crit. Care Med..

[2] Wick K.D., Ware L.B., Matthay M.A. (2024). Acute respiratory distress syndrome. BMJ.

[3] Force A.D.T., Ranieri V.M., Rubenfeld G.D., Thompson B.T., Ferguson N.D., Caldwell E. (2012). Acute respiratory distress syndrome: the Berlin definition. JAMA.

[4] Bastarache J.A., Blackwell T.S. (2009). Development of animal models for the acute respiratory distress syndrome. Dis. Model. Mech..

[5] Gunther A., Walmrath D., Grimminger F., Seeger W. (2001). Pathophysiology of acute lung injury. Semin. Respir. Crit. Care Med..

[6] Dushianthan A., Grocott M.P.W., Murugan G.S., Wilkinson T.M.A., Postle A.D. (2023). Pulmonary surfactant in adult ARDS: current perspectives and future directions. Diagnostics (Basel).

[7] Baer B., Souza L.M.P., Pimentel A.S., Veldhuizen R.A.W. (2019). New insights into exogenous surfactant as a carrier of pulmonary therapeutics. Biochem. Pharmacol..

[8] Baer B., Veldhuizen E.J.A., Possmayer F., Yamashita C., Veldhuizen R. (2018). The wet bridge transfer system: a novel tool to assess exogenous surfactant as a vehicle for intrapulmonary drug delivery. Discov. Med..

[9] Castillo-Sanchez J.C., Cruz A., Perez-Gil J. (2021). Structural hallmarks of lung surfactant: lipid-protein interactions, membrane structure and future challenges. Arch. Biochem. Biophys..

[10] Hamvas A. (2006). Inherited surfactant protein-B deficiency and surfactant protein-C associated disease: clinical features and evaluation. Semin. Perinatol..

[11] Possmayer F., Zuo Y.Y., Veldhuizen R.A.W., Petersen N.O. (2023). Pulmonary surfactant: a mighty thin film. Chem. Rev..

[12] Olmeda B., Garcia-Alvarez B., Gomez M.J., Martinez-Calle M., Cruz A., Perez-Gil J. (2015). A model for the structure and mechanism of action of pulmonary surfactant protein B. FASEB J..

[13] Baoukina S., Tieleman D.P. (2011). Lung surfactant protein SP-B promotes formation of bilayer reservoirs from monolayer and lipid transfer between the interface and subphase. Biophys. J..

[14] Chang R., Nir S., Poulain F.R. (1998). Analysis of binding and membrane destabilization of phospholipid membranes by surfactant apoprotein B. Biochim. Biophys. Acta.

[15] Alonso A., Olmeda B., Perez-Gil J. (2025). Surfactant protein SP-B: one ring to rule the molecular and biophysical mechanisms of the pulmonary surfactant system. Biophys. Rev..

[16] Nag K., Munro J.G., Inchley K., Schurch S., Petersen N.O., Possmayer F. (1999). SP-B refining of pulmonary surfactant phospholipid films. Am. J. Physiol..

[17] Perez-Gil J. (2022). A recipe for a good clinical pulmonary surfactant. Biomed. J..

[18] Enhorning G., Holm B.A. (1993). Disruption of pulmonary surfactant's ability to maintain openness of a narrow tube. J. Appl. Physiol..

[19] Olmeda B., Martinez-Calle M., Perez-Gil J. (2017). Pulmonary surfactant metabolism in the alveolar airspace: biogenesis, extracellular conversions, recycling. Ann. Anat..

[20] De Luca D., Lopez-Rodriguez E., Minucci A., Vendittelli F., Gentile L., Stival E. (2013). Clinical and biological role of secretory phospholipase A2 in acute respiratory distress syndrome infants. Crit. Care.

[21] Seeds M.C., Grier B.L., Suckling B.N., Safta A.M., Long D.L., Waite B.M. (2012). Secretory phospholipase A2-mediated depletion of phosphatidylglycerol in early acute respiratory distress syndrome. Am. J. Med. Sci..

[22] Schmidt R., Markart P., Ruppert C., Wygrecka M., Kuchenbuch T., Walmrath D. (2007). Time-dependent changes in pulmonary surfactant function and composition in acute respiratory distress syndrome due to pneumonia or aspiration. Respir. Res..

[23] Schmidt R., Ruppert C., Markart P., Lubke N., Ermert L., Weissmann N. (2004). Changes in pulmonary surfactant function and composition in bleomycin-induced pneumonitis and fibrosis. Toxicol. Appl. Pharmacol..

[24] Hallman M., Spragg R., Harrell J.H., Moser K.M., Gluck L. (1982). Evidence of lung surfactant abnormality in respiratory failure. Study of bronchoalveolar lavage phospholipids, surface activity, phospholipase activity, and plasma myoinositol. J. Clin. Invest..

[25] Gregory T.J., Longmore W.J., Moxley M.A., Whitsett J.A., Reed C.R., Fowler A.A. (1991). Surfactant chemical composition and biophysical activity in acute respiratory distress syndrome. J. Clin. Invest..

[26] Postle A.D., Clark H.W., Fink J., Madsen J., Koster G., Panchal M. (2022). Rapid phospholipid turnover after surfactant nebulization in severe COVID-19 infection: a randomized clinical trial. Am. J. Respir. Crit. Care Med..

[27] Letsiou E., Htwe Y.M., Dudek S.M. (2021). Secretory phospholipase A(2) enzymes in acute lung injury. Cell Biochem. Biophys..

[28] Kitsiouli E., Nakos G., Lekka M.E. (2009). Phospholipase A2 subclasses in acute respiratory distress syndrome. Biochim. Biophys. Acta.

[29] Attalah H.L., Wu Y., Alaoui-El-Azher M., Thouron F., Koumanov K., Wolf C. (2003). Induction of type-IIA secretory phospholipase A2 in animal models of acute lung injury. Eur. Respir. J..

[30] Touqui L., Arbibe L. (1999). A role for phospholipase A2 in ARDS pathogenesis. Mol. Med. Today.

[31] Neidlinger N.A., Hirvela E.R., Skinner R.A., Larkin S.K., Harken A.H., Kuypers F.A. (2005). Postinjury serum secretory phospholipase A2 correlates with hypoxemia and clinical status at 72 hours. J. Am. Coll. Surg..

[32] Murakami M., Sato H., Taketomi Y. (2023). Modulation of immunity by the secreted phospholipase A(2) family. Immunol. Rev..

[33] Khan S.A., Ilies M.A. (2023). The phospholipase A2 superfamily: structure, isozymes, catalysis, physiologic and pathologic roles. Int. J. Mol. Sci..

[34] Murakami M., Yamamoto K., Miki Y., Murase R., Sato H., Taketomi Y. (2016). The roles of the secreted phospholipase A(2) gene family in immunology. Adv. Immunol..

[35] Munoz N.M., Meliton A.Y., Meliton L.N., Dudek S.M., Leff A.R. (2009). Secretory group V phospholipase A2 regulates acute lung injury and neutrophilic inflammation caused by LPS in mice. Am. J. Physiol. Lung Cell Mol. Physiol..

[36] Seeds M.C., Jones K.A., Duncan Hite R., Willingham M.C., Borgerink H.M., Woodruff R.D. (2000). Cell-specific expression of group X and group V secretory phospholipases A(2) in human lung airway epithelial cells. Am. J. Respir. Cell Mol. Biol..

[37] Granata F., Nardicchi V., Loffredo S., Frattini A., Ilaria Staiano R., Agostini C. (2009). Secreted phospholipases A(2): a proinflammatory connection between macrophages and mast cells in the human lung. Immunobiology.

[38] Nevalainen T.J., Haapamaki M.M., Gronroos J.M. (2000). Roles of secretory phospholipases A(2) in inflammatory diseases and trauma. Biochim. Biophys. Acta.

[39] Nakos G., Kitsiouli E., Hatzidaki E., Koulouras V., Touqui L., Lekka M.E. (2005). Phospholipases A2 and platelet-activating-factor acetylhydrolase in patients with acute respiratory distress syndrome. Crit. Care Med..

[40] Masuda S., Murakami M., Mitsuishi M., Komiyama K., Ishikawa Y., Ishii T. (2005). Expression of secretory phospholipase A2 enzymes in lungs of humans with pneumonia and their potential prostaglandin-synthetic function in human lung-derived cells. Biochem. J..

[41] Hite R.D., Seeds M.C., Safta A.M., Jacinto R.B., Gyves J.I., Bass D.A. (2005). Lysophospholipid generation and phosphatidylglycerol depletion in phospholipase A(2)-mediated surfactant dysfunction. Am. J. Physiol. Lung Cell Mol. Physiol..

[42] Hite R.D., Seeds M.C., Jacinto R.B., Grier B.L., Waite B.M., Bass D.A. (2005). Lysophospholipid and fatty acid inhibition of pulmonary surfactant: non-enzymatic models of phospholipase A2 surfactant hydrolysis. Biochim. Biophys. Acta.

[43] Kulkarni H.S., Lee J.S., Bastarache J.A., Kuebler W.M., Downey G.P., Albaiceta G.M. (2022). Update on the features and measurements of experimental acute lung injury in animals: an official American thoracic Society workshop report. Am. J. Respir. Cell Mol. Biol..

[44] Bartlett G.R. (1959). Phosphorus assay in column chromatography. J. Biol. Chem..

[45] Enhorning G. (1977). Pulsating bubble technique for evaluating pulmonary surfactant. J. Appl. Physiol. Respir. Environ. Exerc. Physiol..

[46] Bligh E.G., Dyer W.J. (1959). A rapid method of total lipid extraction and purification. Can. J. Biochem. Physiol..

[47] Hite R.D., Seeds M.C., Bowton D.L., Grier B.L., Safta A.M., Balkrishnan R. (2005). Surfactant phospholipid changes after antigen challenge: a role for phosphatidylglycerol in dysfunction. Am. J. Physiol. Lung Cell Mol. Physiol..

[48] Hite R.D., Seeds M.C., Jacinto R.B., Balasubramanian R., Waite M., Bass D. (1998). Hydrolysis of surfactant-associated phosphatidylcholine by mammalian secretory phospholipases A2. Am. J. Physiol..

[49] Amidon B., Brown A., Waite M. (1996). Transacylase and phospholipases in the synthesis of bis(monoacylglycero)phosphate. Biochemistry.

[50] Fine J.B., Sprecher H. (1982). Unidimensional thin-layer chromatography of phospholipids on boric acid-impregnated plates. J. Lipid Res..

[51] Irvin C.G., Bates J.H. (2003). Measuring the lung function in the mouse: the challenge of size. Respir. Res..

[52] McGovern T.K., Robichaud A., Fereydoonzad L., Schuessler T.F., Martin J.G. (2013). Evaluation of respiratory system mechanics in mice using the forced oscillation technique. J. Vis. Exp..

[53] Grove L.M., Southern B.D., Jin T.H., White K.E., Paruchuri S., Harel E. (2014). Urokinase-type plasminogen activator receptor (uPAR) ligation induces a raft-localized integrin signaling switch that mediates the hypermotile phenotype of fibrotic fibroblasts. J. Biol. Chem..

[54] Baer B., Putz N.D., Riedmann K., Gonski S., Lin J., Ware L.B. (2023). Liraglutide pretreatment attenuates sepsis-induced acute lung injury. Am. J. Physiol. Lung Cell Mol. Physiol..

[55] Martinez-Calle M., Olmeda B., Dietl P., Frick M., Perez-Gil J. (2018). Pulmonary surfactant protein SP-B promotes exocytosis of lamellar bodies in alveolar type II cells. FASEB J..

[56] Rouser G., Siakotos A.N., Fleischer S. (1966). Quantitative analysis of phospholipids by thin-layer chromatography and phosphorus analysis of spots. Lipids.

[57] Spragg R.G., Lewis J.F., Wurst W., Hafner D., Baughman R.P., Wewers M.D. (2003). Treatment of acute respiratory distress syndrome with recombinant surfactant protein C surfactant. Am. J. Respir. Crit. Care Med..

[58] Beppu O.S., Clements J.A., Goerke J. (1983). Phosphatidylglycerol-deficient lung surfactant has normal properties. J. Appl. Physiol. Respir. Environ. Exerc. Physiol..

[59] De Luca D., Minucci A., Piastra M., Cogo P.E., Vendittelli F., Marzano L. (2012). Ex vivo effect of varespladib on secretory phospholipase A2 alveolar activity in infants with ARDS. PLoS One.

[60] Abraham E., Naum C., Bandi V., Gervich D., Lowry S.F., Wunderink R. (2003). Efficacy and safety of LY315920Na/S-5920, a selective inhibitor of 14-kDa group IIA secretory phospholipase A2, in patients with suspected sepsis and organ failure. Crit. Care Med..

[61] De Luca D., Vendittelli F., Trias J., Fraser H., Minucci A., Gentile L. (2013). Surfactant and varespladib co-administration in stimulated rat alveolar macrophages culture. Curr. Pharm. Biotechnol..

[62] Kennedy B.P., Payette P., Mudgett J., Vadas P., Pruzanski W., Kwan M. (1995). A natural disruption of the secretory group II phospholipase A2 gene in inbred mouse strains. J. Biol. Chem..

[63] Autilio C., Echaide M., Shankar-Aguilera S., Bragado R., Amidani D., Salomone F. (2020). Surfactant injury in the early phase of severe meconium aspiration syndrome. Am. J. Respir. Cell Mol. Biol..

[64] Murphy R.C., Lai Y., Nolin J.D., Aguillon Prada R.A., Chakrabarti A., Novotny M.V. (2021). Exercise-induced alterations in phospholipid hydrolysis, airway surfactant, and eicosanoids and their role in airway hyperresponsiveness in asthma. Am. J. Physiol. Lung Cell Mol. Physiol..

[65] Seidner S.R., Jobe A.H., Ikegami M., Pettenazzo A., Priestley A., Ruffini L. (1988). Lysophosphatidylcholine uptake and metabolism in the adult rabbit lung. Biochim. Biophys. Acta.

[66] Quintero O.A., Wright J.R. (2000). Metabolism of phosphatidylglycerol by alveolar macrophages in vitro. Am. J. Physiol. Lung Cell Mol. Physiol..

[67] Seeger W., Stohr G., Wolf H.R., Neuhof H. (1985). 1985. Alteration of surfactant function due to protein leakage: special interaction with fibrin monomer. J. Appl. Physiol..

[68] Zuo Y.Y., Veldhuizen R.A., Neumann A.W., Petersen N.O., Possmayer F. (2008). Current perspectives in pulmonary surfactant--inhibition, enhancement and evaluation. Biochim. Biophys. Acta.

[69] Nag K., Rodriguez-Capote K., Panda A.K., Frederick L., Hearn S.A., Petersen N.O. (2004). Disparate effects of two phosphatidylcholine binding proteins, C-reactive protein and surfactant protein A, on pulmonary surfactant structure and function. Am. J. Physiol. Lung Cell Mol. Physiol..

[70] Gunther A., Ruppert C., Schmidt R., Markart P., Grimminger F., Walmrath D. (2001). Surfactant alteration and replacement in acute respiratory distress syndrome. Respir. Res..

[71] Seehase M., Collins J.J., Kuypers E., Jellema R.K., Ophelders D.R., Ospina O.L. (2012). New surfactant with SP-B and C analogs gives survival benefit after inactivation in preterm lambs. PLoS One.

[72] Henderson W.R., Chen L., Amato M.B.P., Brochard L.J. (2017). Fifty years of research in ARDS. Respiratory mechanics in acute respiratory distress syndrome. Am. J. Respir. Crit. Care Med..

[73] Rosas L.E., Doolittle L.M., Joseph L.M., El-Musa H., Novotny M.V., Hickman-Davis J.M. (2021). Postexposure liponucleotide prophylaxis and treatment attenuates acute respiratory distress syndrome in influenza-infected mice. Am. J. Respir. Cell Mol. Biol..

[74] Hiansen J.Q., Keating E., Aspros A., Yao L.J., Bosma K.J., Yamashita C.M. (2015). Cholesterol-mediated surfactant dysfunction is mitigated by surfactant protein A. Biochim. Biophys. Acta.

[75] Vockeroth D., Gunasekara L., Amrein M., Possmayer F., Lewis J.F., Veldhuizen R.A. (2010). Role of cholesterol in the biophysical dysfunction of surfactant in ventilator-induced lung injury. Am. J. Physiol. Lung Cell Mol. Physiol..

[76] Al-Saiedy M., Pratt R., Lai P., Kerek E., Joyce H., Prenner E. (2018). Dysfunction of pulmonary surfactant mediated by phospholipid oxidation is cholesterol-dependent. Biochim. Biophys. Acta Gen. Subj..

