# Supplementary material for: Hydrolysis and depletion of phosphatidylglycerol at peak murine acute lung injury
Source: J Lipid Res. 2026 Jun 27;67(7):101068. doi: 10.1016/j.jlr.2026.101068 (PMC13321027; doi:10.1016/j.jlr.2026.101068)
Supplement: Supplemental Table S1 [file mmc1.docx]

**Supplemental Table 1.** Comparison of individual parameters of total lung histology injury score in BALB/c mice intratracheally instilled with either lipopolysaccharide (LPS; 9 mg/kg) or sterile saline (Sham).

| Parameter of Lung Histology Injury Score | Baseline  0 Hours  (N=3) | Sham  48 Hours  (N=3) | LPS  48 Hours  (N=3) | LPS  240 Hours  (N=3) |
| --- | --- | --- | --- | --- |
| Inflammation |  | | | |
| Mean (SD) | 1.61 (0.54) | 1.11 (0.10) | 2.78 (0.54) **^a,b,c^** | 1.56 (0.25) |
| Septal Thickening |  |  |  |  |
| Mean (SD) | 1.22 (0.35) | 0.94 (0.25) | 2.06 (0.10) **^a,b,c^** | 1.11 (0.10) |
| Edema |  |  | | |
| Mean (SD) | 0.17 (0.17) | 0.06 (0.10) | 1.39 (0.38) **^a,b,c^** | 0.28 (0.19) |
| RBCs in Alveolar Space |  |  | | |
| Mean (SD) | 0.28 (0.19) | 0.44 (0.38) | 1.56 (0.98) | 0.33 (0.17) |
| Total Injury Score |  |  | | |
| Mean (SD) | 3.28 (0.82) | 2.56 (0.79) | 7.78 (0.35) **^a,b,c^** | 3.28 (0.63) |
| Definition of Abbreviations: SD = standard deviation, RBC = red blood cells, ^a^p<0.05 vs 0 hours, ^b^p<0.05 vs Sham, ^c^ p<0.05 vs LPS 240 Hours. | | | | |

**Supplemental Figures**

**Supplemental Figure 1**. Pulmonary neutrophil recruitment and illness severity induced by intratracheal (IT) LPS (9 mg/kg) in BALB/c mice. Compared to baseline and sham mice, LPS instilled mice had increased BAL neutrophils (A) and lower body weights (B; 4-136) at 24 to 96 hours post instillation (C; 4-136). No differences were observed between LPS and sham mice across any of these outcomes at baseline, 4-hour, or 240-hour time point. However, saline instillation resulted in lower body weights compared to baseline mice at 24 hours post instillation. Each data point represents the mean of individual mice, with error bars indicating standard error of the mean. ^†^p<0.05 vs baseline (0 hours) and *p<0.05 vs Sham. [Statistical analysis: Two-way ANOVA + Sidak's multiple comparisons test (A-B)]. BAL = bronchoalveolar lavage, IT = intratracheal, LPS = lipopolysaccharide.

**Supplemental Figure 2**. Impaired lung mechanics induced by intratracheal (IT) LPS (9 mg/kg) in BALB/c mice. At 48 hours post instillation, LPS exposed mice displayed increased hysteresis (A; N = 3-20), respiratory system resistance (B; N = 3-20), respiratory system elastance (C; N = 3-20) and tissue elastance (D; N = 3-20) compared to sham and baseline. Saline instillation also increased these parameters at 48 and 96 hours post instillation compared to baseline. No differences were observed between treatment groups across any of the time points for airway resistance (E; N = 3-20) or inspiratory capacity (F; N = 3-20). Each data point represents the mean of individual mice, with error bars indicating standard error of the mean. ^†^p<0.05 vs baseline (0 hours) and *p<0.05 vs Sham. [Statistical analysis: Two-way ANOVA + Sidak's multiple comparisons test (A-F)]. IT = intratracheal, LPS = lipopolysaccharide, Rrs = respiratory system resistance, Ers = respiratory system elastance, H = tissue elastance, Rn = airway resistance, IC = inspiratory capacity.

**Supplemental Figure 3**. Representative whole lung sections in BALB/c mice used for histology scoring. Lungs were stained with H&E and images of the entire section were taken at 20x using a slide scanner. Black brackets indicate regions of interalveolar septal thickening, blue arrows indicate inflammatory cells, red circles indicate RBCs in the airspace, and black circles indicate edema. RBC = red blood cells.

**Supplemental Figure 4.** IT LPS (9 mg/kg) in BALB/c mice does not alter the relative proportion of major surfactant phospholipids in the LA surfactant subfraction. No differences were observed at peak injury or recovery time points with respect to the percentage of PE (A; N = 3-7), SPH (B; N = 3-7), LPC (C; N = 3-7), or PI (D; N = 3-7) comprising the LA surfactant subfraction. However, compared to saline both saline and LPS instillation resulted in higher relative PI compared to baseline at 48 and 96 hours post instillation. Each data point represents the mean of pooled samples, with error bars indicating standard error of the mean. ^†^p<0.05 vs baseline (0 hours) and *p<0.05 vs Sham. [Statistical analysis: Two-way ANOVA + Sidak's multiple comparisons test (A-D)]. IT = intratracheal, LPS = lipopolysaccharides, PL = phospholipids, LA = large aggregates, PE = phosphatidylethanolamine, SPH = sphingomyelin, LPC = lysophosphatidylcholine, PI = phosphatidylinositol.

**Supplemental Figure 5.** IT LPS (9 mg/kg) in BALB/c mice does not alter the absolute amount of major surfactant phospholipids recovered in the LA. No differences were observed at peak injury or recovery time points with respect to the amount of LA PE (A; N = 3-7). SPH (B; N = 3-7), LPC (C; N = 3-7), or PI (D; N = 3-7). However, compared to baseline, saline instillation increased the amount of LA PE recovered at 48 hours post instillation. Both saline and LPS instillation also resulted in more LA SPH recovered at 48 hours compared to baseline. Each data point represents the mean of pooled samples, with error bars indicating standard error of the mean. ^†^p<0.05 vs baseline (0 hours) and *p<0.05 vs Sham. [Statistical analysis: Two-way ANOVA + Sidak's multiple comparisons test (A-D)]. IT = intratracheal, LPS = lipopolysaccharides, PL = phospholipids, LA = large aggregates, PE = phosphatidylethanolamine, SPH = sphingomyelin, LPC = lysophosphatidylcholine, PI = phosphatidylinositol.

**Supplemental Figure 6.** Varespladib treatment of mice after a 48-hour LPS insult did not increase the amount of surfactant recovered nor the relative proportion of major surfactant phospholipids in the LA surfactant subfraction. No differences were observed between varespladib and untreated LPS mice in BAL (A; N = 3-5) or LA (B; N = 3-5) phospholipids. At 4-hours post treatment, LA isolated from Varespladib and untreated LPS mice also had similar percentages of PC (C; N = 3-5), PI (D; N = 3-5), SPH (E; N = 3-5), LPC (F; N = 3-5), and PE (G; N = 3-5). Each data point represents pooled animals, while the horizontal line indicates median. [Statistical analysis: Mann-Whitney test (A-L)]. IT = intratracheal, LPS = lipopolysaccharides, LA = large aggregates, PC = phosphatidylcholine, PI = phosphatidylinositol, SPH = sphingomyelin, LPC = lysophosphatidylcholine.

**Supplemental Figure 7.** Varespladib treatment of mice after a 48-hour LPS insult did not increase the absolute amount of major surfactant phospholipids in the LA. At 4-hours post treatment, LA isolated from Varespladib and untreated LPS mice has similar absolute amounts of PC (A; N = 3-5) PI (B; N = 3-5), SPH (C; N = 3-5), LPC (D; N = 3-5), and PE (E; N = 3-5). Each data point represents pooled animals, while the horizontal line indicates median. [Statistical analysis: Mann-Whitney test (A-L)]. IT = intratracheal, LPS = lipopolysaccharides, LA = large aggregates, PC = phosphatidylcholine, PI = phosphatidylinositol, SPH = sphingomyelin, LPC = lysophosphatidylcholine.

**References**
