# Supplementary material for: Hydrolysis and depletion of phosphatidylglycerol at peak murine acute lung injury
Source: J Lipid Res. 2026 Jun 27;67(7):101068. doi: 10.1016/j.jlr.2026.101068 (PMC13321027; doi:10.1016/j.jlr.2026.101068)
Supplement: Supplemental Data [file mmc2.pdf]

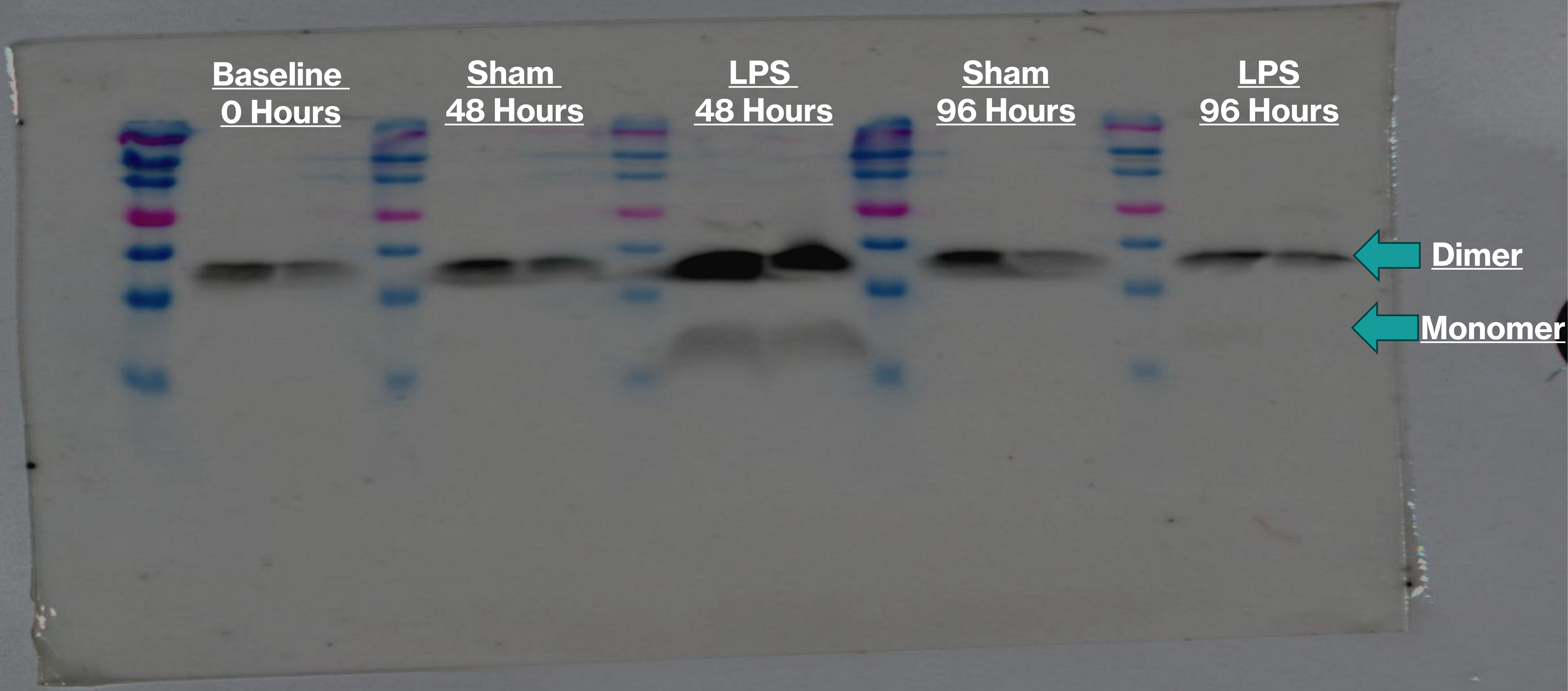

Uncut SP-B Blot 1

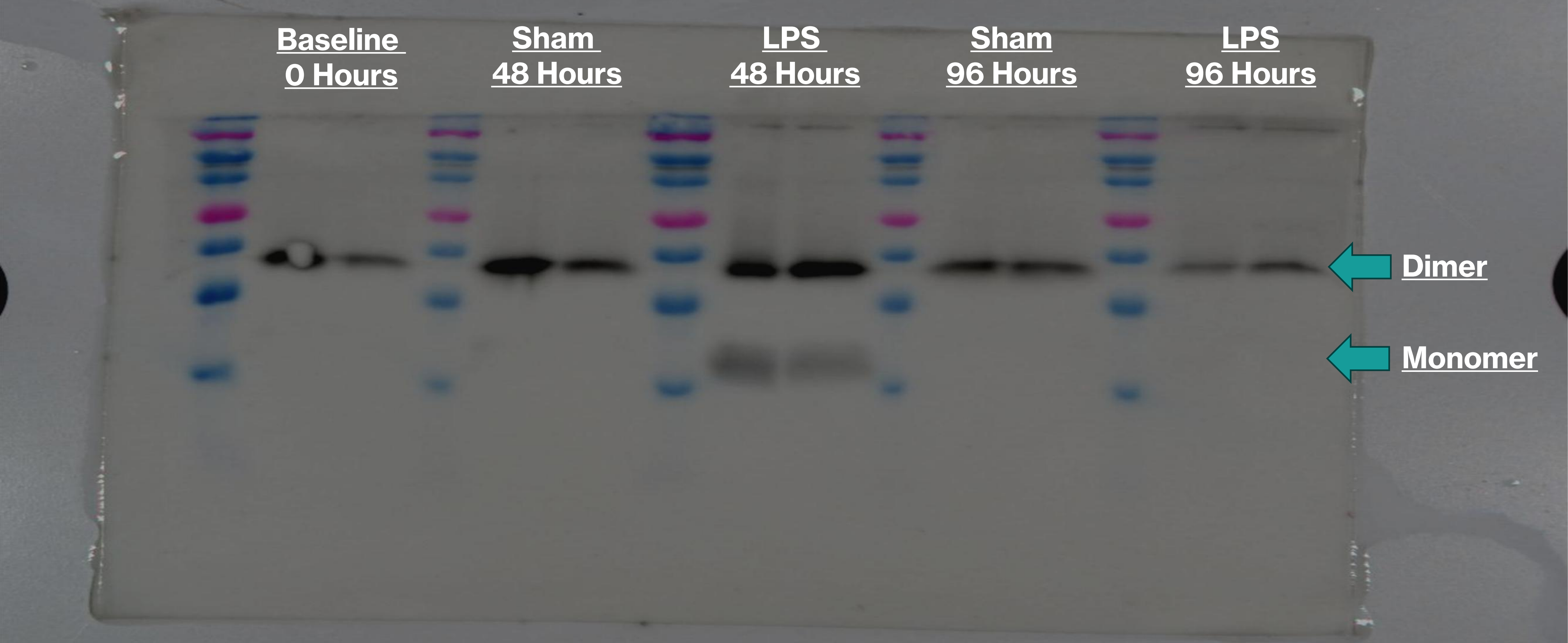

Uncut SP-B Blot 2 : Used as Representative Blot

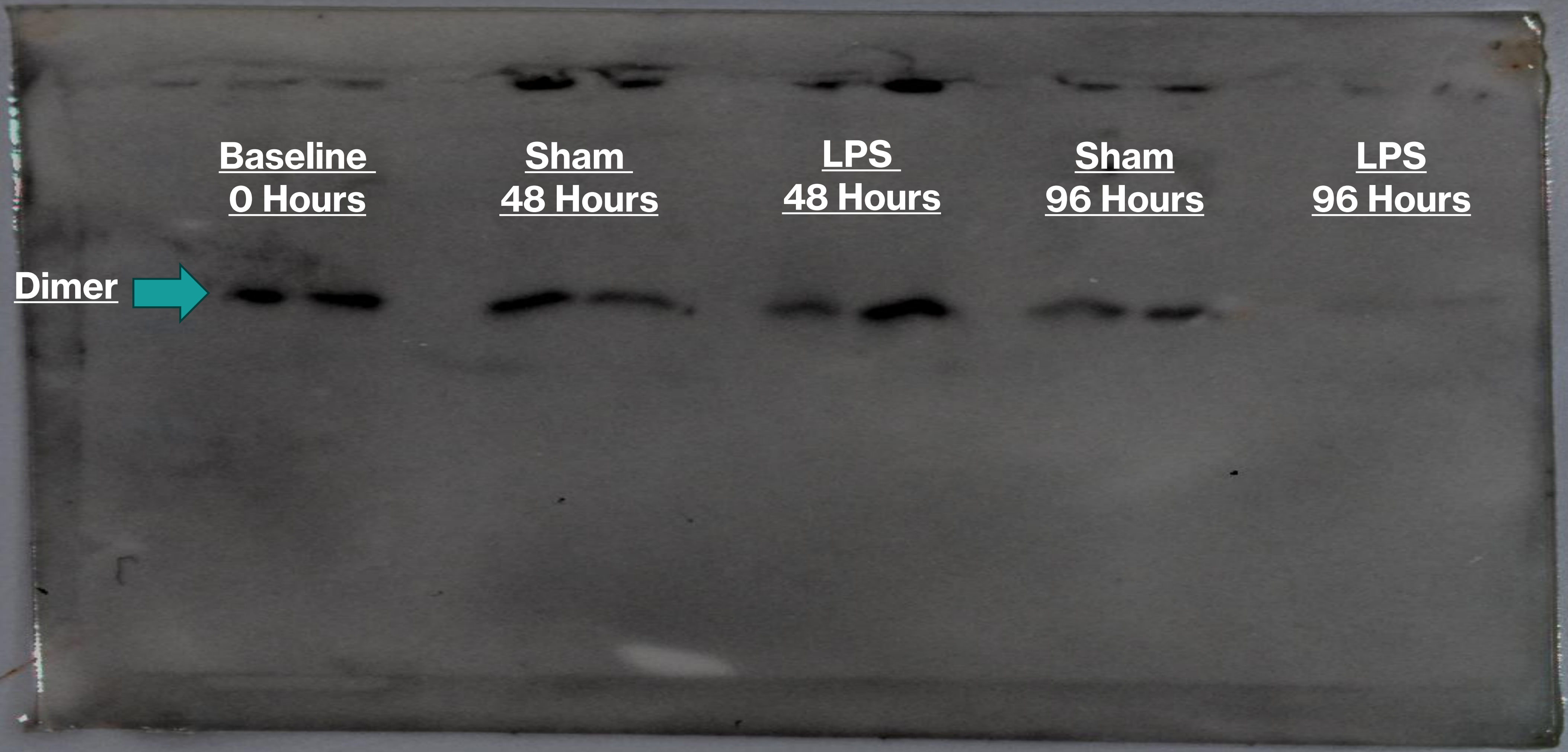

Uncut SP-B Blot 3

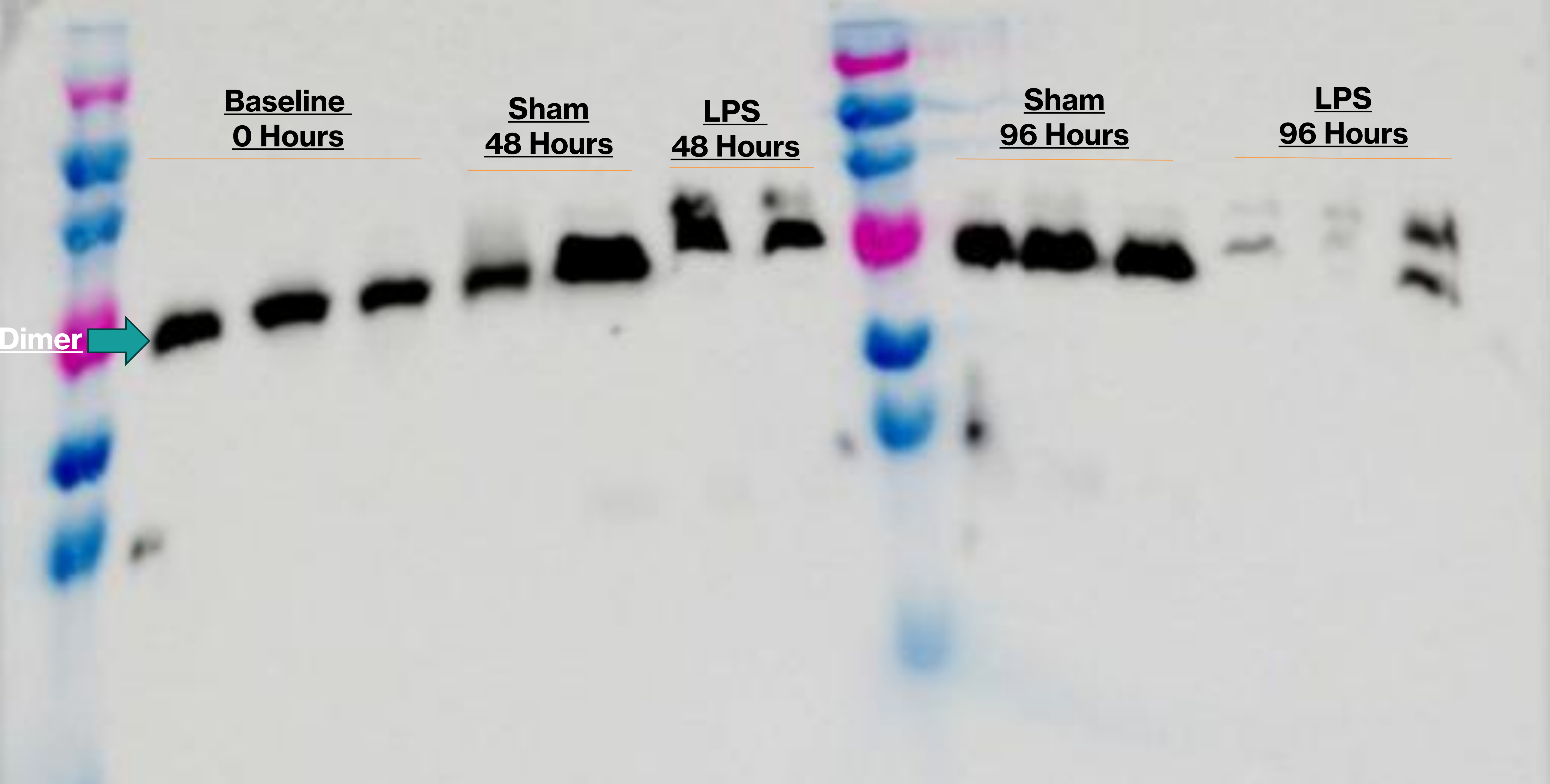

Uncut SP-B Blot 4

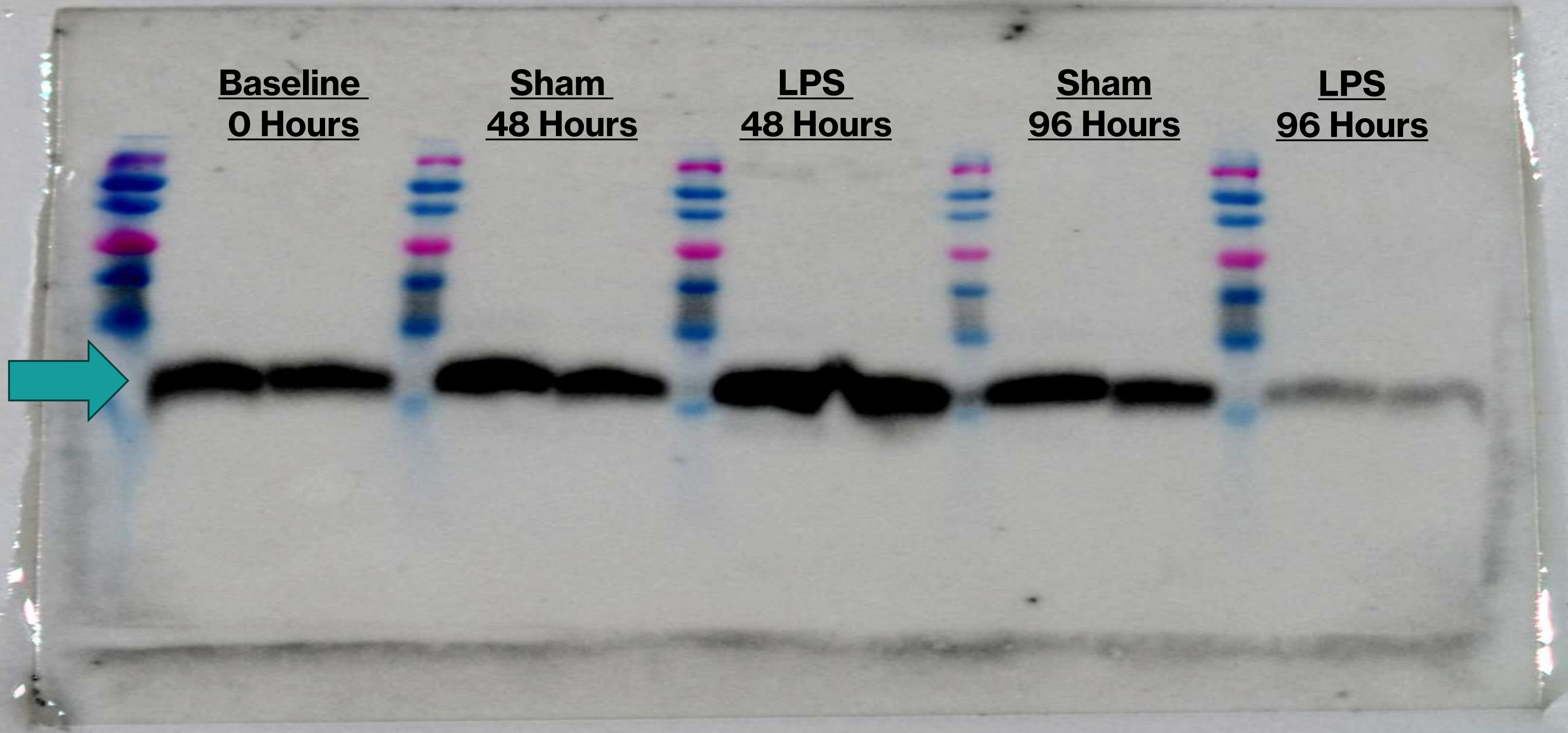

Uncut SP-C Blot 1

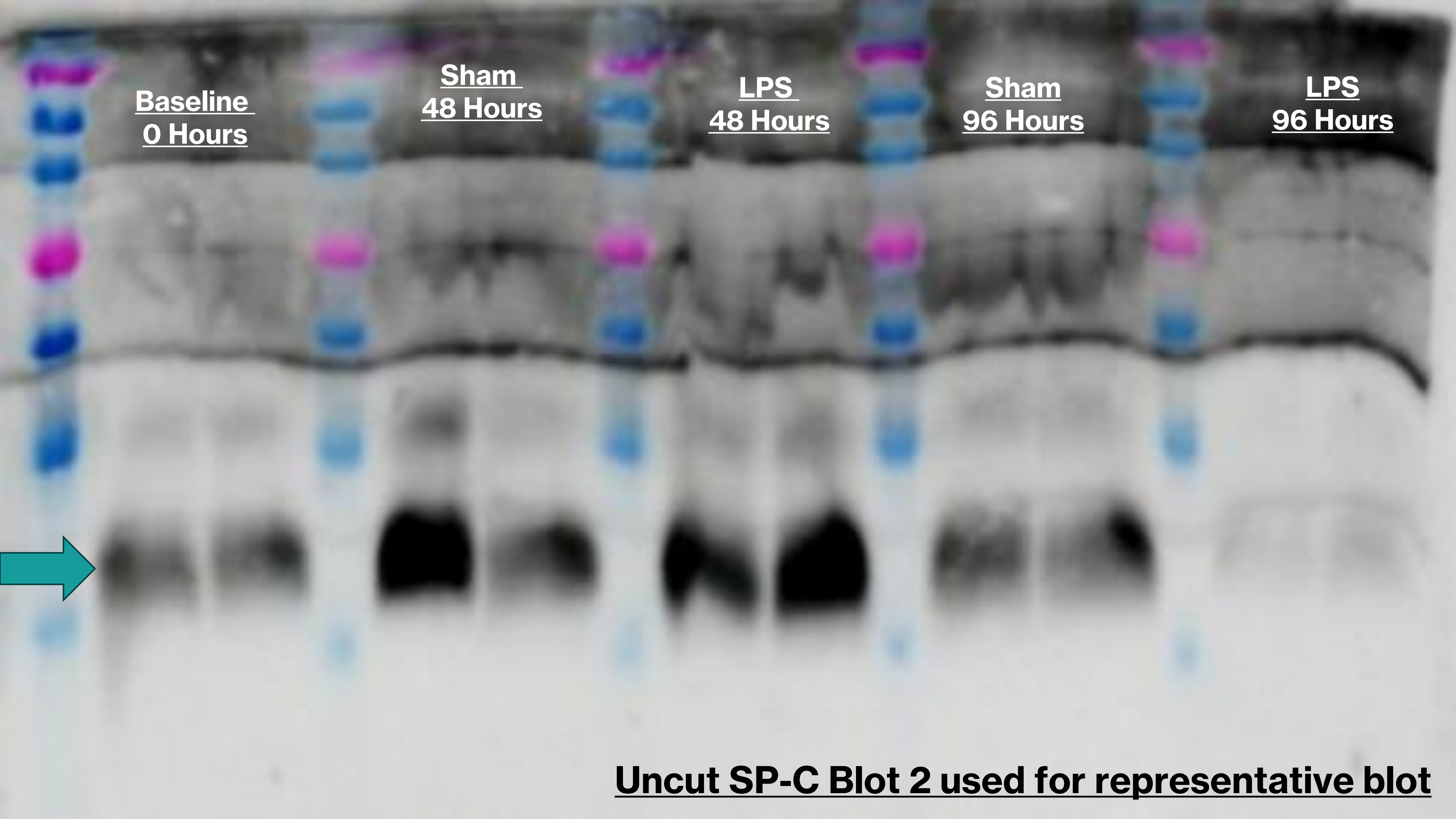
Baseline
0 Hours
Sham
48 Hours
LPS
48 Hours
Sham
96 Hours
LPS
96 Hours
Uncut SP-C Blot 2 used for representative blot

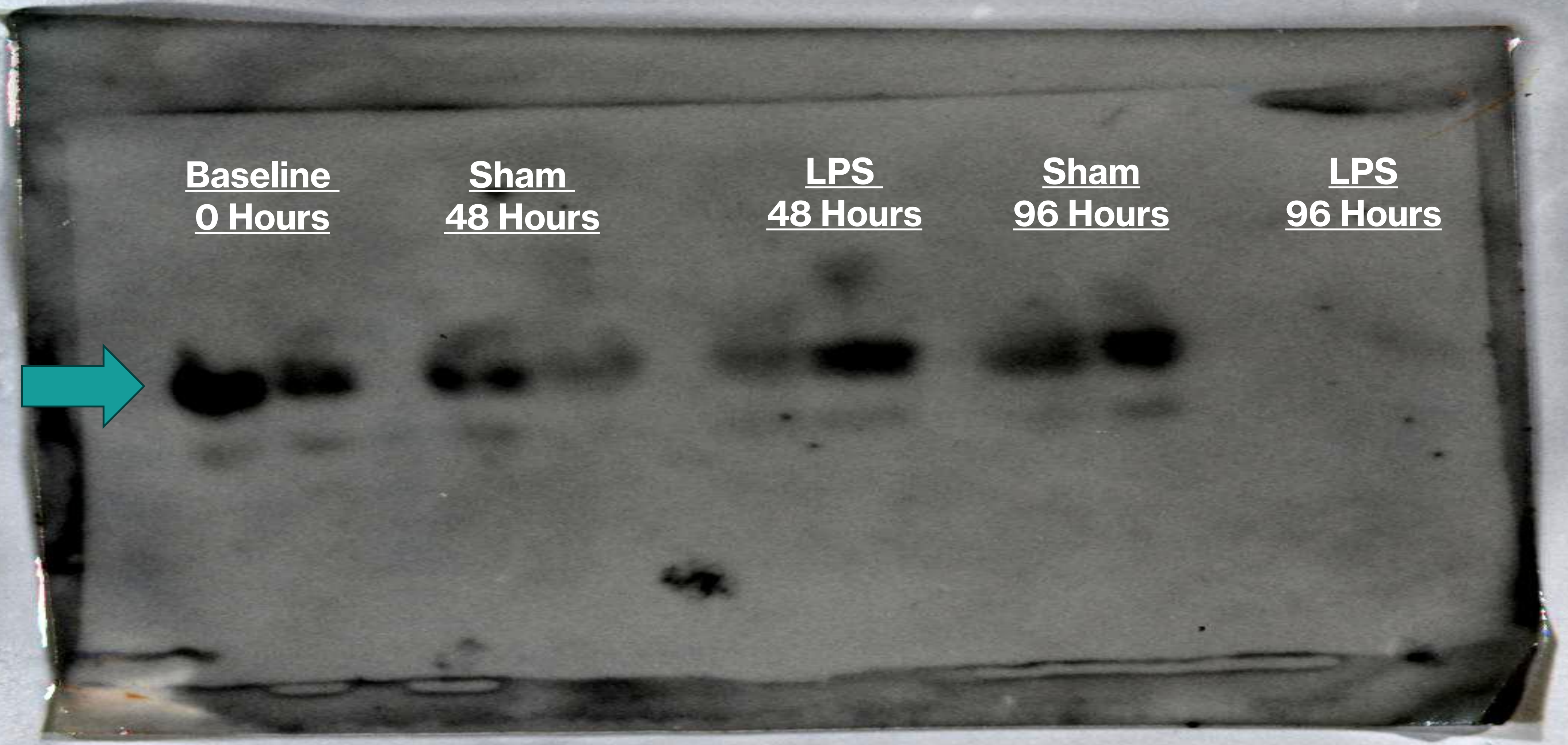

Uncut SP-C Blot 3

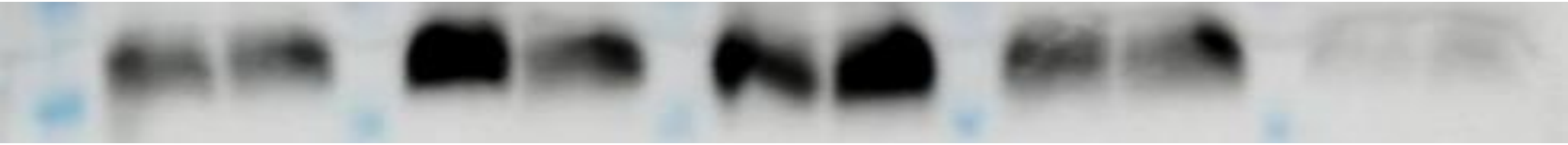

Blot 2 SP-C

Cropped Images for Figure 5

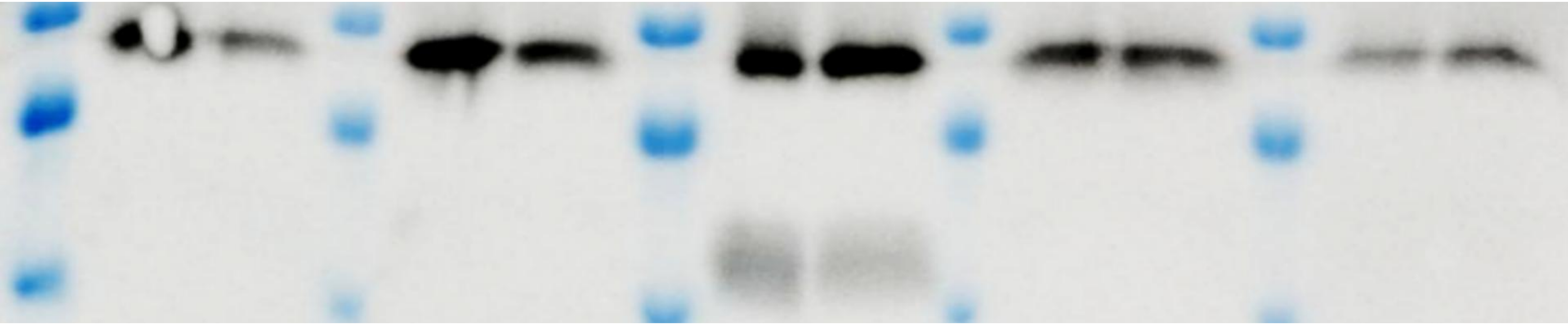

Blot 2 SP-B
